# The fabrication and assessment of mosquito repellent cream for outdoor protection

**DOI:** 10.1038/s41598-022-06185-9

**Published:** 2022-02-09

**Authors:** Hemanga Hazarika, Harshita Krishnatreyya, Varun Tyagi, Johirul Islam, Neelutpal Gogoi, Danswrang Goyary, Pronobesh Chattopadhyay, Kamaruz Zaman

**Affiliations:** 1 Division of Pharmaceutical Technology, Defence Research Laboratory, Tezpur, Assam 784001 India; 2 Girijananda Chowdhury Institute of Pharmaceutical Science, Dekargaon, Tezpur, Assam 784501 India; 3 Eurofins Agroscience Services Pvt. Ltd., Tirupur, Tamil Nadu 641603 India; 4 Coromandel Int. Ltd., Shameerpet, Telangana 500101 India; 5 Department of Pharmaceutical Sciences, Dibrugarh University, Dibrugarh, Assam 786004 India

**Keywords:** Biological techniques, Chemical biology, Drug discovery, Ecology, Molecular biology, Plant sciences

## Abstract

Mosquito-borne infections like dengue, malaria, chikungunya, etc. are a nuisance and can cause profound discomfort to people. Due to the objectional side effects and toxicity associated with synthetic pyrethroids, *N*,*N*-diethyl-3-methylbenzamide (DEET), *N*,*N*-diethyl phenylacetamide (DEPA), and *N*,*N*-di ethyl benzamide (DEBA) based mosquito repellent products, we developed an essential oil (EO) based mosquito repellent cream (EO-MRC) using clove, citronella and lemongrass oil. Subsequently, a formulation characterization, bio-efficacy, and safety study of EO-MRC were carried out. Expression of Anti-OBP2A and TRPV1 proteins on mosquito head parts were studied by western blotting. In-silico screening was also conducted for the specific proteins. An FT-IR study confirmed the chemical compatibility of the EOs and excipients used in EO-MRC. The thermal behaviour of the best EOs and their mixture was characterized by thermogravimetric analysis (TGA). GC–MS examination revealed various chemical components present in EOs. Efficacy of EO-MRC was correlated with 12% *N*,*N*-diethyl benzamide (DEBA) based marketed cream (DBMC). Complete protection time (CPT) of EO-MRC was determined as 228 min. Cytotoxicity study on L-132 cell line confirmed the non-toxic nature of EO-MRC upon inhalation. Acute dermal irritation study, acute dermal dose toxicity study, and acute eye irritation study revealed the non-toxic nature of EO-MRC. Non-target toxicity study on *Danio rerio* confirmed EO-MRC as safer for aquatic non-target animals. A decrease in the concentration of acetylcholinesterase (AChE) was observed in transfluthrin (TNSF) exposed Wistar rats. While EO-MRC did not alter the AChE concentrations in the exposed animals. Results from western blotting confirmed that Anti-OBP2A and TRPV1 proteins were inhibited in TNSF exposed mosquitoes. Mosquitoes exposed to EO-MRC showed a similar expression pattern for Anti-OBP2A and TRPV1 as the control group. In silico study revealed eight identified compounds of the EOs play significant roles in the overall repellency property of the developed product. The study emphasizes the mosquito repellent activity of EO-MRC, which could be an effective, eco-friendly, and safer alternative to the existing synthetic repellents for personal protection against mosquitoes during field conditions.

## Introduction

Mosquitoes are the key vectors of several tropical diseases including malaria, filariasis, and viral diseases such as dengue, chikungunya, west-Nile, yellow fever and zika^1–4^. Dengue spread by *Aedes aegypti* (L.) and *Aedes albopictus* (Skuse), is closely associated with human residences in metropolises^5–7^. Mosquito borne infections have been documented to be a principal contributing cause to illness amongst long-term expatriates, travellers, and military units deployed overseas, especially in tropical and sub-tropical areas^8^. Various major and minor epidemics amongst the US and foreign military have been recorded when deployed to malaria-endemic areas of West Africa, North Africa, the South Pacific and the China–Burma–India borders during World War II, the Korean War, and Vietnam Conflict^9–11^.

Regrettably, there are neither vaccines nor specific treatments for most mosquito-borne diseases^12,13^. Hence, the population of disease endemic countries are strongly recommended to avoid mosquito bites, primarily by wearing appropriate clothing and by applying topical repellents on the exposed body parts^14–16^. Prevention of mosquito bites in indoor spaces can be achieved by utilizing long lasting insecticidal nets (LLINs) and indoor residual spray (IRS), a flagship recommendation by the World Health Organization (WHO). Mosquito repellent electrical vaporizer formulations are effective in reducing mosquito densities in indoor areas. Repellents in the form of cream, gel and lotions are typically applied to the exposed skin to get protection from mosquito bites in outdoor conditions^17^. Topical repellents tend to be low-volatility compounds^18^ providing a vapor barrier over the skin, or slowly evaporating into the ambient air and warding off the arthropod^19,20^. Convection currents caused by air drafts and limb motion of the host can reduce the vapours over the exposed skin^18^. Repellent compounds with a high boiling point are not suitable for the preparation of topical formulations due to their insufficient evaporation rate, whereas components having low boiling points dissipate quickly and easily^21^. *N*,*N*-Diethyl-3-methylbenzamide (DEET), *N*,*N*-diethyl phenylacetamide (DEPA), dimethyl phthalate (DMP), *N*,*N*-di ethyl benzamide (DEBA) and allethrin, are utilized in most of the commercial mosquito repellent formulations^11,22^, which are persistent synthetic chemicals, and non-biodegradable^23^. Their higher exposure to the environment may hamper the ecosystem^23–25^. DEET can dissolve plastic on eyeglasses, wrist watches etc.; having a strong smell, creating oily and burning sensations, and may even cause discomfort, particularly when applied at higher doses^26^. Additionally, emerging problems related to development of resistance, side effects, toxicity to non-target organisms, and ecological concerns, amongst these synthetic repellent agents are causing serious concerns^27^. Users, therefore, prefer repellents containing alternate actives^28^.

Herbal essential oils (EOs) have various chemical components, which possess insecticidal, repellent and deterrent properties^29^. Various chemical compositions present in EOs contribute to the efficacy of topical mosquito repellent formulations. Studies suggest that EOs might be almost as effective as DEET^29–31^. The Centre for Disease Control and Prevention, United States, recommends oil of lemon and eucalyptus as an alternative to DEET due to its efficacy against insects^32^. International organizations like the Environmental Protection Agency (EPA), United States (US) and the World Health Organization (WHO), as well as other countries approved several EOs for their diversified use with regards to their accessibility, availability, reliability, economic and low risk assessment with complex chemistry and are not subject to federal registration requirements^27,33^. Numerous natural EO based products are in use as insect repellents. However, plant products have been registered as safe and effective topical insect repellents^34^, but their allergenicity, mutagenicity, genotoxicity and complete protection time are still questionable. Extensive research on effective, safe and environmentally friendly mosquito repellent topical formulation for outdoor protection with extended protection time have been increasing gradually^35^. Therefore, in the present research, we screened fourteen EOs for their repellent activity against *Aedes albopictus* mosquitoes, and developed an EO based longer lasting mosquito repellent cream (EO-MRC) formulation utilizing the best blend of EOs viz. citronella oil, clove oil and lemongrass oil. A characterization, efficacy, safety and toxicity study of the EO-MRC was carried out with a Silico molecular docking study to summarize the binding affinity of the EO components to the target site. We also studied the expression of Anti-OBP2A and TRPV1 antibody in a mosquito's head part by western blotting, which might play a significant role in repelling mosquitoes from the exposed skin of a host. In summary, the main objective of our study is to develop and characterize an EO based topical cream formulation for mosquito repellent with reduced toxicity for outdoor protection.

## Results

### Screening of EOs

Percentage mosquito repellency of fourteen EOs revealed their efficacy against *Aedes albopictus* (*Ae. Albopictus*). However, maximum response was evident with citronella oil (95.83%), clove oil (91.66%), lavender oil (86.95%) and lemongrass oil (95.83%), under K&D module bioassay. Lemon grass oil and citronella oil exhibited maximum repellency whereas basil oil elicited least repellency (29.16%). In this study, four best oils among the fourteen essential oils were further evaluated for their synergistic effect against mosquitoes under the K&D module. The blend of clove oil, citronella oil and lemongrass oil exhibited maximum repellency property (96%) while the blend of lemon grass oil, lavender oil and clove oil exhibited least response (80%). The percentage repellency for different concentrations of fourteen EOs and blends of the best oils along with their ED_50_ and ED_95_ are shown in Table 1.

**Table 1:** Screening of essential oils and their blends (best four oils) for mosquito repellency under K&D module.

Essential oils and their blends	Percentage of mosquito repellency with respect to applied doses of essential oils (mg/cm^2^)			ED_50_ (mg/cm^2^)	ED_95_ (mg/cm^2^)
	0.04 mg/cm^2^	0.004 mg/cm^2^	0.0004 mg/cm^2^		
Basil	29.16	08.69	04.34	0.35	155.75
Bergamot	37.50	17.39	04.34	0.33	347.11
Camphor	52.17	25.00	13.04	0.09	236.30
Cinnamon	43.47	29.16	08.69	0.07	238.5
Citronella	95.83	78.26	52.17	0.0004	0.0413
Levender	86.95	78.26	45.83	0.0004	0.1709
Eucalyptus	75.00	60.86	34.78	0.0018	2.09
Jasmine	39.13	20.83	04.00	0.0862	20.37
Clove	91.66	75.00	54.16	0.0003	0.0686
Lemongrass	95.83	82.60	56.52	0.0002	0.0313
Mentha	39.13	20.83	04.34	0.0885	23.14
Patchouli	34.78	20.83	08.69	0.1542	221.78
Rosemarry	60.86	58.33	30.43	0.0025	1.76
Wild turmeric	87.50	65.21	26.08	0.0018	0.1195
CNL + CLV	90.00	63.63	45.83	0.0007	0.0811
CNL + LMG	90.90	69.56	39.13	0.0005	0.1004
CNL + LVDR	86.36	60.86	43.47	0.0012	0.3215
CLV + LVDR	91.66	69.56	52.17	0.0004	0.1544
CLV + LMG	92.00	73.91	60.86	0.0002	0.1743
LVDR + LMG	84.00	70.83	52.17	0.0003	0.9152
CNL + CLV + LMG	96.00	92.00	62.50	0.0001	0.0156
CNL + LVDR + LMG	88.00	76.00	58.33	0.0001	0.0358
CNL + LVDR + CLV	92.00	79.16	62.50	0.0001	0.0352
LMG + LVDR + CLV	80.00	70.83	58.33	0.0001	13.16

*CNL* citronella, *CLV* clove, *LVDR* lavender, *LMG* lemon grass.

### Fourier transform-infra red spectroscopy (FT-IR)

Chemical compatibility of EOs and excipients to be used in EO-MRC was evaluated by FT-IR spectroscopy. The FT-IR spectra of analytes are presented in Supplementary Fig. S1, respectively.

Citronella oil showed characteristic peak at 791/cm, 1380/cm, and 1460/cm for C–H bending which might be due to the presence of 1,2-disubstituted, alkane and aldehyde group. A peak at 1260/cm might be due to the presence of alkyl aryl ether, another peak at 1666/cm and 1714/cm might be due to the presence of α, β-unsaturated ester, conjugated ketone. Peak at 1844/cm and 2908/cm might be due to the presence of alkane and another characteristic peak at 3425/cm corresponds to alcoholic group. A peak at 990/cm for C=C bending; 1260/cm for C–O stretching; 1714/cm and 1666/cm for C=O stretching; 2844/cm and 2908/cm C–H stretching; 3425/cm for O–H stretching respectively.

Clove oil showed peak at 752/cm for C–H bending; 1308/cm for S=O stretching; 1714/cm for C=O stretching; 2932/cm for C–H stretching; 3472/cm for O–H stretching respectively.

Lemon grass oil showed peak at 791/cm for C–H bending; 1030/cm for S=O stretching; 1284/cm for S=O stretching; 1515/cm N–O stretching; 1610/cm for C=C stretching; 1746/cm for C=O stretching; 2932/cm for C–H stretching respectively.

Glycerol myristate showed peak at 728/cm and 1465/cm for C–H bending; 1181/cm for C–O stretching; 1730/cm for C=O stretching; 2844/cm and 2940/cm for C–H stretching 3322/cm for N–H stretching respectively.

Cetyl alcohol showed sharp peak at 735/cm and 1465/cm for C-H bending; 1069/cm for C–O stretching, which might be due to the presence of primary alcohol; 2836/cm and 2908/cm for C–H stretching, might be due to the presence of alkane; 3234/cm for O–H stretching; 3337/cm for N–H stretching respectively.

Stearic acid showed peak at 754/cm for C-H bending; 1284/cm for C–O stretching; 1730/cm for C=O stretching; 2971/cm for C–H stretching 3449/cm for O–H stretching respectively.

Vanillin showed peak at 735/cm for C–H bending, which might be due to some monosubstituted functional groups; 1260/cm for C–O stretching, might be due to the presence of aromatic ester group; another peak at 1515/cm N–O stretching; 1595/cm and 1658/cm for C=C stretching; 3218/cm for O–H stretching respectively.

EDTA showed peak at 776/cm for C–H bending; 995/cm for C=C bending; 1269/cm for C–O stretching; 1412/cm for S=O stretching; 1595/cm for N–H bending; 3218/cm for O–H stretching respectively.

Methyl paraben exhibited peak at 960/cm for C=C bending; 1465/cm for C–H bending; 1730/cm for C=O stretching; 2916/cm and 3281/cm for C–H stretching respectively.

Sodium benzoate showed peak at 831/cm for C–H bending; 1061/cm for C–O stretching, which might be due to the presence of primary alcohol; 1412/cm for O–H bending; 1547/cm for N–O stretching; 1603/cm for C=C stretching; 3027/cm and 3059/cm for C–H stretching; 3624/cm for O–H stretching respectively.

Light liquid paraffin showed peak at 1388/cm for O–H bending; 1460/cm for C–H bending; 2852/cm and 2955/cm C–H stretching respectively.

Isopropyl myristate showed peak at 1093/cm for C–O stretching; 1380/cm for O–H bending; 1467/cm for C–H bending; 1738/cm for C=O stretching; 2860/cm and 2923/cm for C–H stretching; 3457/cm for O–H stretching respectively.

Dimethicone showed peak at 1061/cm for C–O stretching; 1260/cm for C–O stretching; 1412/cm for O–H bending; 1929/cm for C–H bending; 2916/cm and 2971/cm for C–H stretching respectively.

Vitamin E showed peak at 743/cm for C–H bending; 1770/cm for C=O stretching; 2868/cm for C–H stretching; 3504/cm for O–H stretching respectively.

Rosemary oil showed peak at 982/cm for C=C bending 1465/cm for C–H bending 1750/cm for C=O stretching 2923/cm for C–H stretching respectively.

Polyethylene glycol showed peak at 1308/cm for C–O stretching; 1475/cm for C–H bending; 1714/cm for C=O stretching 2844/cm and 2927/cm for C–H stretching respectively.

Tween 20 showed peak at 1117/cm for C–O stretching; 1722/cm for C=O stretching; 2876/cm and 2916/cm for C–H stretching respectively.

Glycerol showed peak at 1110/cm for C–O stretching, which might be due to the presence of secondary alcohol, and a characteristic peak at 1420/cm for O–H bending might be correspondents to alcoholic group respectively.

Propyl paraben showed peak at 960/cm for C=C bending; 1269/cm for C–O stretching; 1436/cm for O–H bending; 1690/cm for C=O stretching; 1937/cm for C–H bending 2964/cm for C–H stretching and peak at 3305/cm for O–H stretching respectively.

Physical mixture exhibited peak at 1085/cm for C–O stretching, which might be due to the presence of primary alcohol; 1167/cm for C–O stretching corresponds to ester group; 1603/cm for C=C stretching due to the presence of conjugated alkene; 1722/cm for C=O stretching; 1921/cm for C–H bending for aromatic compound; 2852/cm and 2947 for C–H stretching; 3600/cm for O–H stretching might be due to the presence of alcoholic group respectively.

FT-IR study confirmed the compatibility of the formulation ingredients. The peaks of the physical mixture were intact and correlated to the individual components and no interaction was observed.

### Thermogravimetric analysis (TGA)

Thermal stability of citronella oil, clove oil, lemon grass oil and their mixture were investigated by TGA and results are presented in Supplementary Fig. S2. Citronella oil showed 98.09% mass loss below 300 ℃ which might be caused by decomposing the organic content of the sample. The weight of clove oil remained constant up to 60 ℃ and started decomposition/mass loss from 60 to 171.7 ℃. At second weight loss region, from 171.7 to 599.6 ℃, clove oil left a residual mass of about 0.87% respectively. Lemon grass oil showed decomposition ranging from 60 to 228 ℃. The blend of citronella, clove and lemon grass oil showed first weight loss region from 30 to 260 ℃, respectively, where all EO components got decomposed. A residual mass of about − 16.18% was remained at 599.6 °C under second weight loss region (228–600 °C). The TGA profile for EO-MRC showed considerable variations in the weight loss behaviour. Within a temperature range from 50 to 260 °C, removal of loaded EO molecules with water might occurred from repellent cream bases. The second weight loss region was evidenced from 270 to 600 °C which might be due to the decomposition of excipients used to formulate EO-MRC.

### Formulation optimization

**Figure 1:**
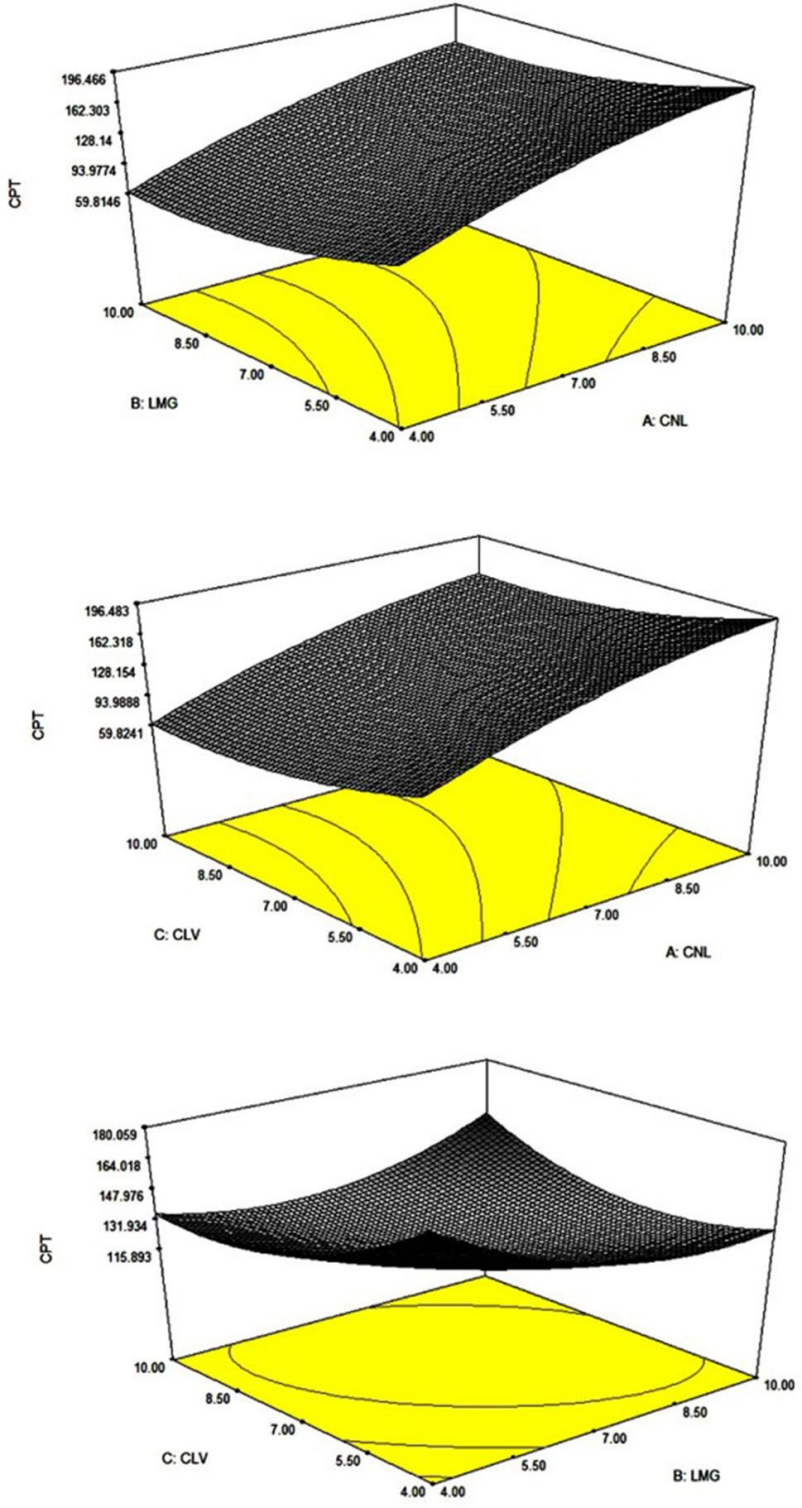
Software generated response surface plot showing effect of the essential oil concentrations (X1, X2) on maximum protection time (Y1). Optimization was done by using Design-Expert software (Version 6.0.8, Stat-Ease Inc., USA, https://www.statease.com/software/design-expert/). The optimum formulation was based on the set criteria of maximum protection time. Where, *CPT* complete protection time, *CLV* clove oil, *LMG* lemon grass oil, *CNL* citronella oil.

Optimization was done by setting the criteria for desirability functions in Design-Expert software. A three-dimensional response surface plot was generated by the software (Fig. 1) which are very useful to study the interaction effects of the factors on the responses. Response surface plots describe the effect of various factors on the response at a time^36^. Here, Fig. 1 indicates the effect of different concentration of EOs on complete protection time (CPT) against mosquitoes. The optimized formula was based on the set criteria of maximum protection time. Therefore, seventeen cream formulations with the predicted levels of formulation factors were prepared. The non-linear computer-generated quadratic model was:

1 $$Y = 102 + 45A - 7.5B - 7.5C - 13.5A^{2} + 16.5B^{2} + 16.5C^{2} + 0.00AB + 0.00AC + 15.00BC$$

where, *‘Y’* was the complete protection time (CPT), the response variable, associated with each factor level combination; and citronella oil (A), lemongrass oil (B) and clove oil (C) were the coded levels (at 2 levels; low and as high level) of dependent variables.

The variables, along with their levels, were selected based on the results from preliminary experimentation are shown in Table 2. The experimental design matrix for the amounts of A, B and C, used to prepare each of the seventeen formulations generated by the software and the responses were observed. ANOVA was performed using the same software to obtain the most effective formulation (Table 3). Nine formulation solutions were generated, following 17-run, 3-factor, 3-level Box–Behnken design (BBD) (Table 4). The best EO-based mosquito repellent cream formulation was found to have the following compositions: 8.13% citronella oil, 4% clove oil, and 4% lemon grass oil, and excipients q.s. to 100% (w/w), and the optimized formula for 50 g EO-MRC are shown in Table 5.

**Table 2:** Design summary and variables in Box Behnken design.

Design summary					
Study type	Response Surface	Experiments	17		
Initial design	Box Behnken	Design model	Quadratic		

Response (independent variable)	Name	Units	Obs	Minimum	Maximum
Y1	CPT	Min	17	60.00	180.00

Factors (dependent variable)	Name	Units	Type	Low level	High level
A	CNL	%	Numeric	4.00	10.00
B	LMG	%	Numeric	4.00	10.00
C	CLV	%	Numeric	4.00	10.00

**Table 3:** Summary of ANOVA for the response parameter (CPT).

**Response**	**CPT**					
ANOVA for response surface quadratic model						
Analysis of variance table [partial sum of squares]						
Source	Sum of squares	DF	Mean square	F value	Prob > F	
Model	20,996.47	9	2332.941	8.247772	0.0055	Significant
A	16,200	1	16,200	57.27273	0.0001	
B	450	1	450	1.590909	0.2476	
C	450	1	450	1.590909	0.2476	
A2	767.3684	1	767.3684	2.712919	0.1435	
B2	1146.316	1	1146.316	4.052632	0.0840	
C2	1146.316	1	1146.316	4.052632	0.0840	
AB	0	1	0	0	1.0000	
AC	0	1	0	0	1.0000	
BC	900	1	900	3.181818	0.1176	
Residual	1980	7	282.8571			
Lack of fit	900	3	300	1.111111	0.4428	Not significant
Pure error	1080	4	270			
Cor total	22,976.47	16

**Table 4:** Nine solutions for formulation was generated by Design-Expert, following 17-run, 3-factor, 3-level Box–Behnken design. CPT against mosquitoes were determined and percentage error was calculated.

Solutions							
Number	CNL (%)	LMG (%)	CLV (%)	Predicted CPT (min)	Experimental CPT (min)	% error	
1	9.68	4.60	4.02	183.67	156	17.73	
2	8.99	4.09	4.29	182.149	150	21.43	
3	10.00	4.12	4.08	192.875	150	28.58	
4	9.62	4.69	4.11	180.177	162	11.22	
5	9.97	4.03	4.45	187.868	162	15.96	
6	9.69	4.17	4.15	188.55	168	12.23	
7	8.13	4.00	4.00	180.009	174	03.45	Selected
8	10.00	10.00	10.00	166.499	144	15.62	
9	9.96	10.00	10.00	166.27	150	10.84

**Table 5:** Optimized formula for EO-MRC.

Formula for 50 g EO-MRC			
Cream bases	Ingredients	Quantity (w/w)	Use
**Oil phase**	**Phase A**		
	Citronella oil	2–4 g	
	Clove oil	2–4 g	Mosquito repellent active ingredients
	Lemon grass oil	2–4 g	
	**Phase B**		
	Glyceryl monostearate	6–8 g	Emulsifier, emollient
	Cetyl alcohol	4–6 g	Stiffening agent/thickener
	Stearic acid	4–7 g	Emulsifier and oil base
	Vanillin	0.5–2 g	Synergist
	Di-sodium EDTA	0.1–0.5 g	Chelating agent
	Methyl parabean	0.1–0.5 g	Oil soluble preservatives
	Sodium benzoate	0.04–0.08 g	Preservative
	Light liquid paraffin	1.5–2.5 g	Emollient, stabilizer
	Isopropyl myristate	0.5–1.5 g	Emollient, thickening agent
	Dimethicone	1.5–3.5 g	Dispersion agent, emulsion stabilizer
	Vitamin E	1.5–2 g	Antioxidant
	Rosemary oil	1.5–2 g	Fragrances/perfumes
**Aqueous phase**	**Phase C**		
	Poly ethylene glycol	4–6 g	Antifreeze, humactant
	Tween 20	1–2 g	Surfactant
	Glycerine	1–4 g	Humectant, plasticizer, antifreeze
	Propyl parabean	0.1–0.4 g	Water soluble preservative
	Demineralised water	q.s.–50 g	Solvent

### Efficacy assessment

**Figure 2:**
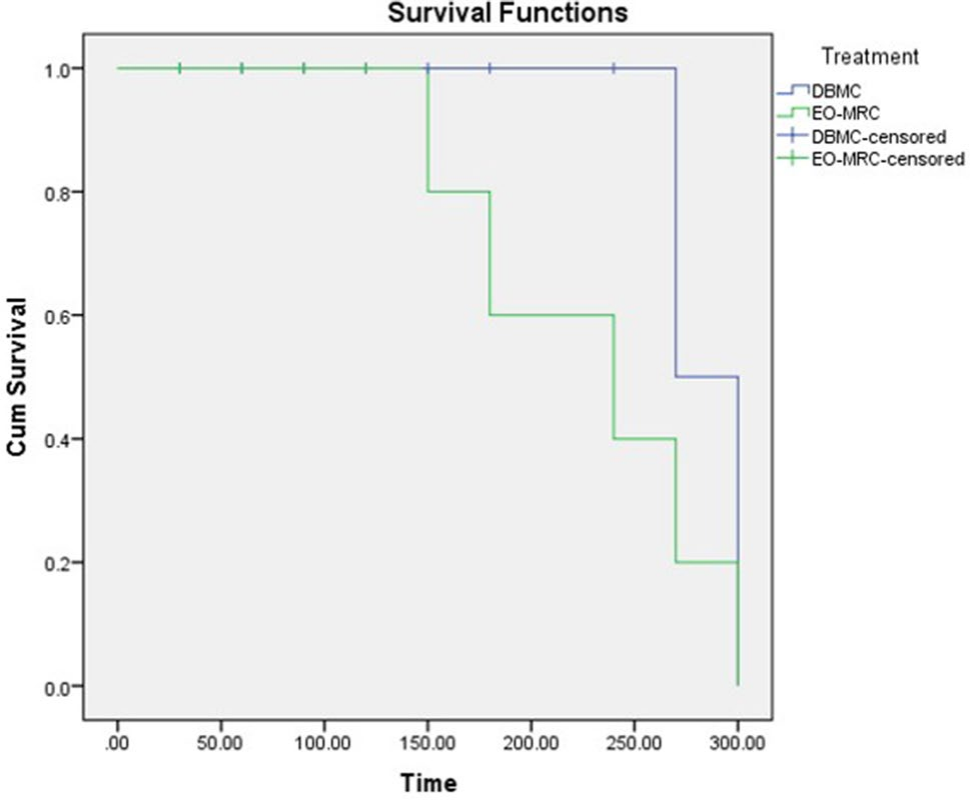
Cumulative Survival vs Time plot against EO-MRC and DBMC to determine complete protection time (CPT). The CPT value was evaluated for EO-MRC and DEBA based marketed cream (DBMC). Datas were evaluated using IBM SPSS Statistics 21 software (version 21.0, 1 New Orchard Road, Armonk, New York 10504-1722, United States, https://www.ibm.com/analytics/spss-statistics-software). Performing the Kaplan–Meier Survival Function, CPT for EO-MRC and DBMC was determined.

EO-MRC showed promising results against *Ae. albopictus*. The CPT value for EO-MRC was evaluated and compared with DBMC. The repellency values were evaluated using IBM SPSS Statistics 21 software. Performing Kaplan–Meier Survival Function. CPT for EO-MRC and DBMC was determined as 228 min and 285 min, respectively. The results are shown in Table 6 and Fig. 2.

**Table 6:** Estimation of complete protection time for EO-MRC and DBMC.

Treatment	Means and medians for survival time					
	Mean^a^				Median	
	Estimate	Std. error	95% confidence interval		Estimate	Std. error
			Lower bound	Upper bound		
DBMC	285.000	15.000	255.600	314.400	270.000	0.00
EO-MRC	228.000	27.821	173.471	282.529	240.000	65.727

^a^Estimation is limited to the largest survival time if it is censored.

### Identification of chemical components

Based on the GC–MS analysis of the 14 EOs, various chemical components were identified, which are known to be the major chemical constituents of those particular oils (Table 7). We reported the GC–MS results for these fourteen EOs in our previous research article^37^. Chemical compositions viz., carene, caryophyllene, citral, d-limonene, betulla oil, camphene, carveol, cinnamaldehyde, caryophyllene, citronellal, citronellol, limonene, eugenol, geraniol, isoneral, linalool, longifolene, l-4-terpeneol and α-terpineol were identified by GC–MS. Here, we carried out molecular docking to focus on the binding affinity of these chemicals (ligands) to the mosquito antennal proteins, which could be the first report on this study area.

**Table 7:** Identified compounds of essential oils (EOs) and the selected blend with retention indices (RI) and relative peak area percentage.

Compounds	RI_Exp_	RI_Lit_	Different EOs and their chemical compounds with retention indices and relative peak area percentage (%)														
			Basil (%)^a^	Bergamot (%)^a^	Camphor (%)^a^	Cinnamon (%)^a^	Citronella (%)^a^	Clove (%)^a^	Eucalyptus (%)^a^	Jasmine (%)^a^	Lavender (%)^a^	Lemon grass (%)^a^	Mentha (%)^a^	Patchouli (%)^a^	Rosemarry (%)^a^	Wild turmeric (%)^a^	Blend* (%)^a^
α-Pinene	935	937	–	–	–	–	–	–	–	–	–	–	–	05.3	–	–	–
Camphene	955	952	–	–	–	–	–	–	13.0	–	–	04.4	06.8	–	09.0	24.8	02.0
β-Pinene	982	979	–	–	–	–	–	–	–	–	23.5	–	06.5	12.8	38.0	–	–
Carene	985	1011	–	09.3	49.0	–	–	–	81.0	–	30.0	–	–	–	85.0	–	–
p-Cymene	1026	1024	–	–	–	–	–	–	–	–	03.0	–	–	–	–	–	–
Limonene	1033	1030	–	100.0	53.0	–	31.6	–	–	–	53.0	–	–	–	–	09.3	–
Eucalyptol	1037	1032	03.5	–	–	–	–	–	100.0	–	49.0	24.0	02.0	–	100.0	13.4	–
γ-Terpinene	1061	1060	–	–	29.3	–	–	–	08.7	–	13.0	–	–	–	01.7	–	–
Fenchone	1100	1096	–	–	18.0	–	–	–	–	–	–	–	–	–	–	–	–
Linalool	1101	1099	42.0	06.5	–	–	16.0	–	–	59.0	69.0	02.6	-	03.0	03.4	–	04.4
Citronellal	1152	1153	–	–	04.0	–	46.0	–	–	–	02.3	–	–	–	–	–	13.4
Isoborneol	1160	1157	–	–	–	–	–	–	–	–	–	–	–	–	10.8	–	–
Borneol	1165	1167	–	–	08.3	–	–	–	–	–	–	–	–	–	–	–	–
Isoneral	1169	1170	–	–	–	–	05.6	–	–	–	–	11.75	–	–	–	10.0	65.0
Terpenen-4-ol	1191	1182	–	–	–	–	–	2.5	–	–	08.2	–	–	–	05.3	–	–
α-Terpineol	1201	1189	–	05.4	–	–	–	–	06.9	16.3	16.2	–	–	–	02.8	02.8	–
Methyl salicylate	1201	1192	–	–	–	02.8	–	03.0	–	1.5	–	–	–	–	–	–	–
Estragole	1202	1196	100.0	05.4	–	–	–	–	–	–	–	–	–	–	–	–	–
Carveol	1219	1219	–	03.5	–	–	–	–	–	–	–	–	–	–	–	–	–
Citronellol	1236	1228	–	–	–	–	15.0	–	–	01.5	–	–	–	–	–	–	07.6
Pulegone	1239	1237	–	–	–	–	–	–	–	–	–	–	02.12	–	–	–	–
d-Carvone	1251	1246	–	04.4	–	–	–	–	–	–	–	–	–	–	–	–	–
Geraniol	1253	1255	01.0	–	–	–	100.0	–	–	–	–	05.2	–	–	–	–	41.4
Geranial	1275	1270	09.3	–	–	–	37.0	–	–	–	–	44.4	–	–	–	–	31.1
Cinnamaldehyde	1277	1274	–	–	–	100.0	–	–	–	–	–	–	–	–	–	–	–
Eugenol	1367	1357	01.0	–	–	–	–	46.0	–	01.0	–	–	–	–	–	–	31.9
Methyl eugenol	1399	1402	02.0	–	–	–	–	–	–	–	–	–	–	–	02.1	–	–
Trans-α-bergamotene	1442	1435	05.0	–	–	–	09.1	–	–	–	–	–	–	–	–	–	–
Patchouli alcohol	1648	1656	–	–	–	–	–	–	–	–	–	–	–	60.0	–	–	–
Aciphyllene	1511	1501	–	–	–	–	–	–	–	–	–	–	–	25.0	–	–	–

RI_Exp._: Experimentally determined linear retention indices using homologous series of C_7_–C_30_ alkanes.

RI_Lit._: Linear retention indices taken from literature [NIST 14 (2014) and Adams (2007)].

Blend*: Mixture (1:1:1) of citronella, lemon grass and clove oil.

(%)^a^ : Relative peak area percentage.

**–:** Compounds not identified.

Reprinted from Acta Tropica, 210 (2020), Hazarika et al., “Essential oil based controlled-release non-toxic evaporating tablet provides effective repellency against Musca domestica”, Pages No. 9, Copyright (2020), with permission from Elsevier.

### % Assay

**Figure 3:**
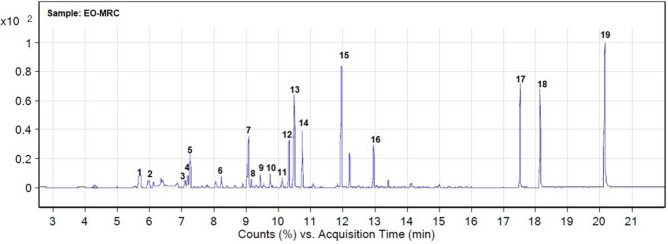
GC–MS chromatogram of EO-MRC; where, 1: 3-carene; 2: camphene; 3: o-cymene; 4: d-limonene; 5: eucalyptol; 6: linalool; 7: citronellal; 8: isoneral; 9: isoborneol; 10: l-alpha-terpineol; 11: citronellol; 12: citral; 13: geraniol; 14: citral; 15: eugenol; 16: caryophyllene; 17: isopropyl myristate; 18: 1-hexadecanol; 19: 1-hexadecanol.

Four points (concentrations) calibration curves were built for eugenol and citronellol (Supplementary Fig. S3). The percentage assay of eugenol and citronellol in EO-MRC was found to be 60.59% and 55.08%, respectively. GC–MS chromatogram of EO-MRC is shown in Fig. 3. Different chemical components of EOs viz., carene, camphene, o-cymene, d-limonene, eucalyptol, linalool, citronellal, isoneral, isoborneol, l-alpha-terpineol, citronellol, citral, geraniol, eugenol, caryophyllene, isopropyl myristate were identified in EO-MRC.

### Physicochemical parameters

EO-MRC and placebo formulation were prepared to obtain a uniform and stable oil in water emulsion with aesthetic appearance. EO-MRC showed a good uniformity and spread ability with acceptable odour and colour. pH of EO-MRC and placebo formulation was found to be 7.3 ± 0.08 and 6.93 ± 0.13 respectively. Values of density (1.02 ± 0.01 g/mL) and viscosity (28,878.33 ± 594.99) indicated sufficient stability of EO-MRC (data are shown in Supplementary Table S1). Further, studies regarding the effect of long-term storage are required to establish the shelf life of the formulations.

### Cytotoxicity by MTT assay

**Figure 4:**
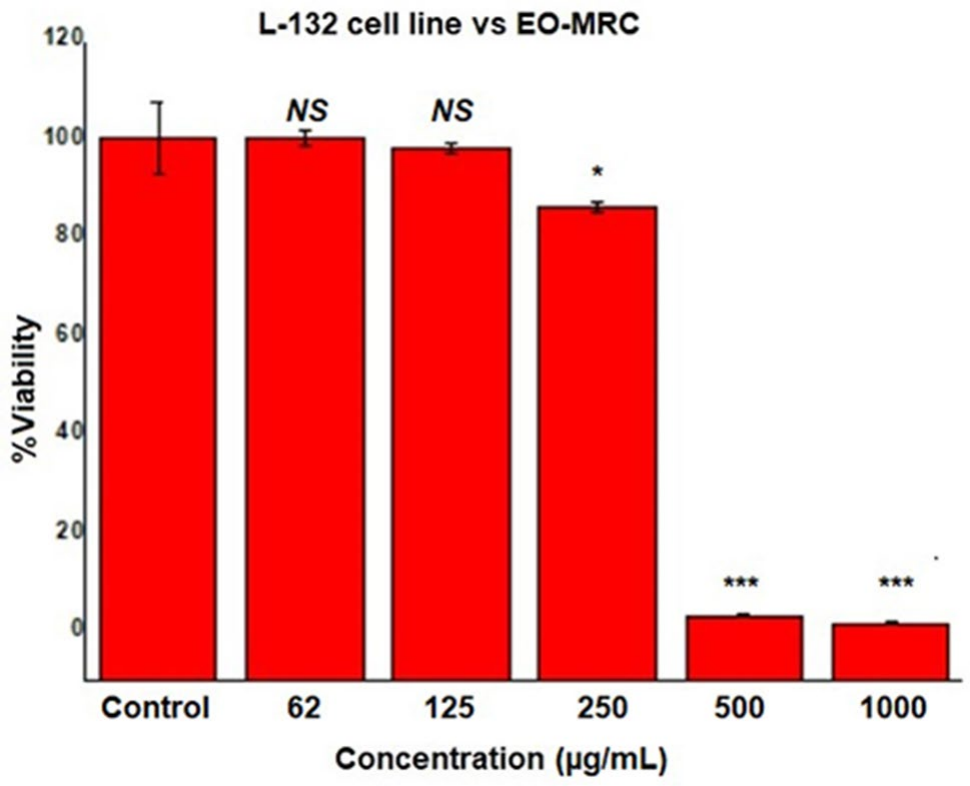
Comparative histogram of cell viability study for EO-MRC at different test concentrations. A decreasing pattern of human lung epithelial cell line (L-132) mortality was observed with increasing concentrations of the EO-MRC. EO-MRC concentration of 500 and 1000 µg/mL was evidenced with significant decrease (p  < 0.001) in cell viability. L-132 was also significantly (p   <  0.05) inhibited by 250 µg/mL of EO-MRC, however, concentration of 62 and 125 µg/mL did not show any significant differences in cell viability as compared to un-treated or control L-132 cell line. ANOVA followed by Dunnett’s multiple comparison tests. Where, *NS* = p > 0.05; *p < 0.05; ***p   <  0.001.

A decreasing pattern of human lung epithelial cell (L-132) mortality was observed with increasing concentrations of the EO-MRC, while there was a noticeable decrease in cell viability after exposure to the different EO-MRC concentrations. Figure 4 represents the estimated cell viability, by MTT assay, to control (untreated) and 62 µg/mL, 125 µg/mL, 250 µg/mL, 500 µg/mL, and 1000 µg/mL, of EO-MRC formulation in L-132 cultures. 500 µg/mL and 1000 µg/mL concentration were evidenced with significant decrease (p  < 0.001) in cell viability, however, 250 µg/mL also inhibited cell viability (p  < 0.05) and EO-MRC concentration of 62 µg/mL and 125 µg/mL did not show any significant differences in cell viability as compared to un-treated L-132 cell lines. ANOVA followed by Dunnett’s multiple comparison tests, where, *NS* = p > 0.05; *p ˂ 0.05; ***p ˂ 0.001 respectively.

### Acute dermal irritation study

In the study, no adverse dermal responses like erythema or oedema were observed after 72 h treatment period in both the placebo and EO-MRC treated rabbit groups. However, rabbits under the positive control group developed severe erythema as well as oedema after 72 h of treatment. The PII value of both the placebo and EO-MRC treated groups were found to be 0, which indicates the non-irritating nature of EO-MRC. Positive control group showed PII value about 5.16, indicates severe irritation as per the standard guidelines (data are available in Supplementary Table S2).

### Repeated dose dermal toxicity study

**Figure 5:**
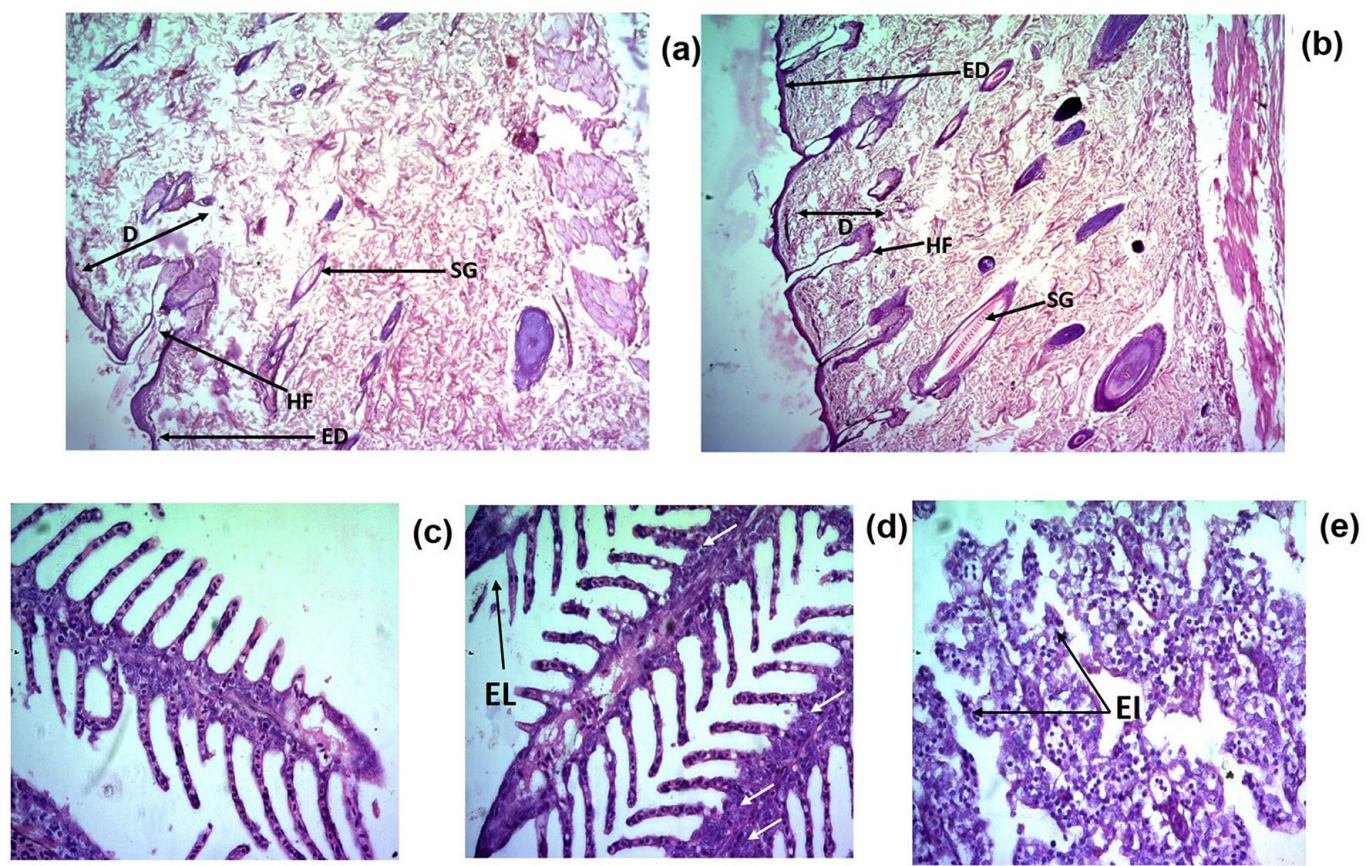
Histopathology of skin tissue under repeated dermal toxicity study for EO-MRC on rats (**a**) control skin; (**b**) EO-MRC treated skin. No remarkable changes had been observed for EO-MRC exposed skin tissues under histopathology study. Histopathology of *D. rerio* gill tissue (**c**) control gill; (**d**) EO-MRC exposed gill; (**e**) deltamethrin exposed gill. EO-MRC exposed gill shows same tissue architecture as control group. DLM exposed (negative control) group shows abnormality in tissue architecture. White arrow: basis hyperplasia; EI: erythrocyte infiltration; EL: epithelial lifting.

No treatment-related clinical signs were observed during the study period. Neither any erythema or oedema was present, nor the treated area of skin was abraded. Aberrations in the locomotor activity were also absent. The daily food and water consumption were normal. All rats had normal body weight, and appeared active and healthy. Histopathological observations of skin evidenced with normal architecture of epidermis (ED), dermis (D), sebaceous gland (SG) and hair follicles (HF) shown in Fig. 5a control skin; Fig. 5b EO-MRC treated skin. Repeated dermal treatment of EO-MRC did not cause any adverse effects on the body weight and feed consumption of the animals as compared to control group. All animals were found to be healthy throughout the study period.

### Acute eye irritation

**Figure 6:**
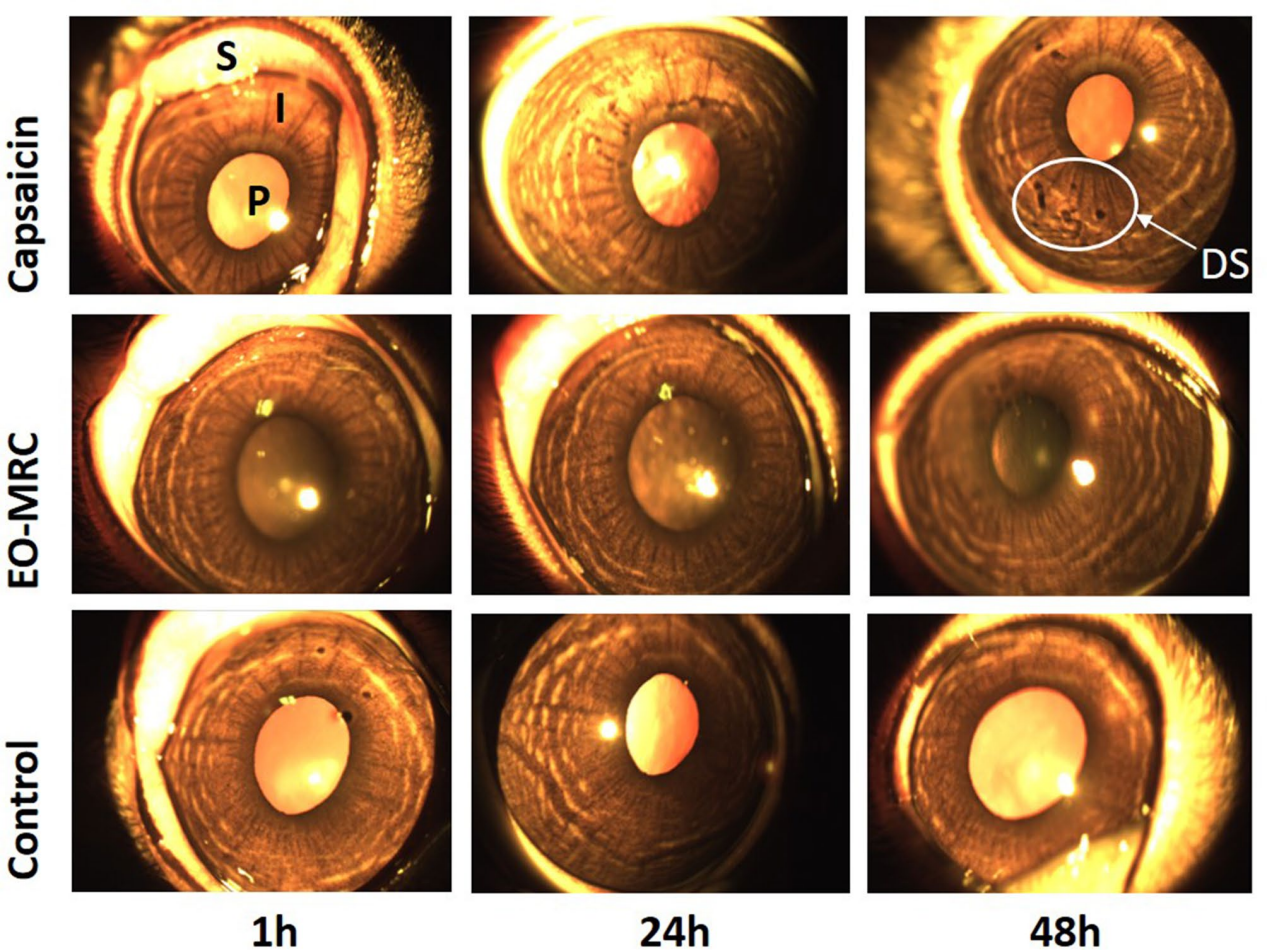
Acute eye irritation study on rabbit eye after 1 h, 24 h, and 48 h of control (untreated), EO-MRC, capsaicin (negative control) exposed groups. Capsaicin was used here as a known irritant, specially used in capsigranade under defence utilization, belonging to the Category 2B, as the reversibility of animal responses. EO-MRC exposed group was evidence with non-irritant to ocular tissues.

From acute eye irritation study on rabbits, our results categorize EO-MRC as non-irritant (Fig. 6). However, capsaicin (negative control group), as a known irritant specially used in defence utilization, was categorized in Category 2B (as per the UN-GHS system), as the irritation responses was observed within 48 h.

### Acute toxicity test on *Danio rerio *(*D. rerio*)

The most frequent pathological modifications were epithelial lifting, erythrocyte infiltration, and basic hyperplasia. Under all observed morphological changes, deltamethrin (DLM) showed more severe effects on gill structure. Minimal toxicity to the gill tissues were observed in EO-MRC exposed group, and was evidenced with erythrocyte infiltration, and basis hyperplasia. Results are shown in Fig. 5c control gill; Fig. 5d EO-MRC exposed gill; Fig. 5e deltamethrin exposed gill.

### AChE activity assay

**Figure 7:**
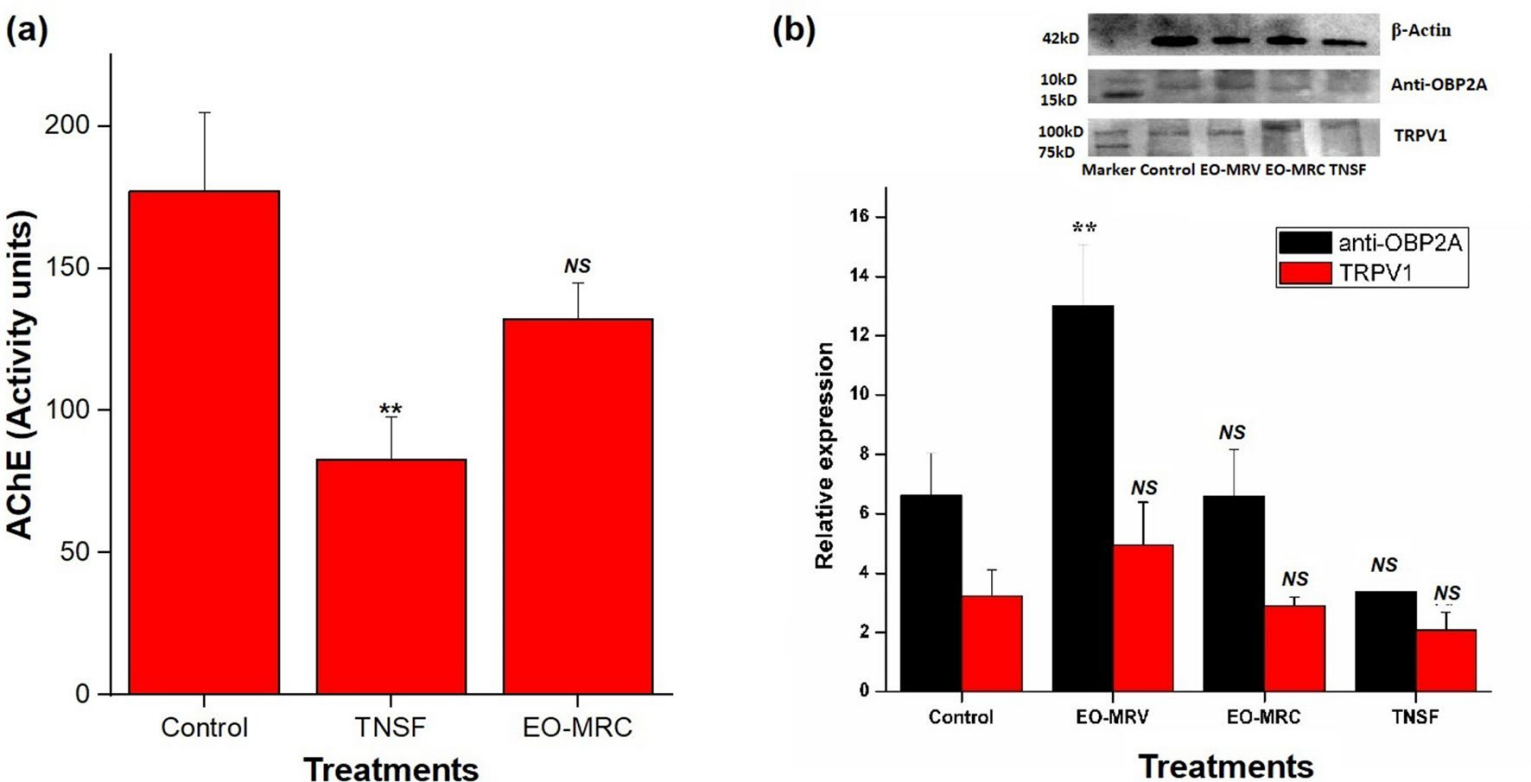
Change in AChE activity may result from exposure to certain chemicals or insecticides, which acts as cholinesterase inhibitors. Most of the EO components have been reported as AChE inhibitors. (**a**) AChE activity assay on wistar rats after TNSF and EO-MRC exposure; significant decrease (p   <  0.01) in AChE was observed in TNSF exposed group. EO-MRC exposed group did not show any significant differences (p > 0.05) in AChE concentrations as compared with the control group; (**b**) relative expression levels of TRPV1 and Anti-OBP2A, in mosquito head part after exposure to TNSF and EO-MRC as quantified by ImageJ. Significant (p   <  0.01) over expression of anti-OBP2A was observed in EO-MRV exposed mosquito head part (13.02 ± 2.05) but, in case of TNSF exposed group, expression level was inhibited (p > 0.05). EO-MRC exposed mosquitoes showed similar expression levels as the control mosquito (p > 0.05). Similar expression pattern has been recorded for TRPV1 also (p  > 0.05). TRPV1 expression was lower in TNSF exposed mosquitoes as compared to the control (p  > 0.05). But, in case of EO-MRV exposed mosquitoes, a higher expression of TRPV1 was observed, however, there was no any significant change (p > 0.05) has been recorded in EO-MRC exposed group of mosquitoes as compared to the control. ANOVA followed by Dunnett's multiple comparison tests. Where, *NS* = p > 0.05; **p   <  0.01. Original blots/gels are presented in Supplementary Figs. S9–Figs. S11.

AChE enzymes are located at the synaptic cleft that hydrolyse the neurotransmitter acetylcholine (ACh) to acetate and choline and terminates synaptic transmission. Changes in AChE activity might results from exposure to certain chemicals or insecticides, which acts as cholinesterase inhibitors. From the values shown in Fig. 7a, it was clear that the activity level of AChE decreased (83 ± 15 units) significantly (p   <  0.01) in transfluthrin (TNSF) exposed rats as compared to control (177 ± 28 units) respectively. In EO-MRC exposed rats, no significant decrease (p > 0.05) in AChE was recorded and the value was found to be 132 ± 13 units respectively. ANOVA followed by Dunnett's multiple comparison tests. Where, *NS* = p > 0.05; **p   <  0.01.

### Protein expression in mosquito head part

#### Protein estimation

Protein concentrations obtained for EO-MRV, EO-MRC, TNSF and control mosquitoes were 7.14 mg/mL, 3.03 mg/mL, 9.146 mg/mL and 3.275 mg/mL, respectively. Based on these concentrations, the amount of each treatment sample containing 50 μg protein was calculated and loaded into the respective SDS PAGE gels. Hence, based on the aforementioned concentrations, 6.68 μL, 16.4 μL, 5.46 μL and 15.26 μL of EO-MRV, EO-MRC, TNSF and control samples were loaded for SDS PAGE respectively.

#### Western blotting

Relative expression levels of TRPV1 and Anti-OBP2A, as quantified by ImageJ, are shown in Fig. 7b. As a control, the housekeeping gene, β-actin, was experimented on prior to blotting of the other antibodies and showed profound expression in the mosquito head part. Expression of anti-OBP2A was shown in 22 kDa, showed its higher expression (p   <  0.01) in EO-MRV exposed mosquito head part (13.02 ± 2.05), but in case of TNSF exposed group, expression level was inhibited (3.37 ± 0.93), (p > 0.05). EO-MRC exposed mosquitoes showed similar expression levels (6.6 ± 1.5) as control mosquito (6.62 ± 1.43), no any significant differences were observed (p  > 0.05). There were no any significant changes (p > 0.05) recorded in EO-MRC exposed group of mosquitoes for TRPV1 (2.9 ± 0.28) as compared to the control. TRPV1 in the mosquito head part showed bands at an expected molecular weight of 100 kDa. In terms of band intensity, WB showed that TRPV1 expression was lower, however it was not significant (p > 0.05) in TNSF exposed mosquitoes (2.07 ± 0.58) as compared to the control samples (3.23 ± 0.88). But, in case of EO-MRV exposed mosquitoes, a higher expression (p > 0.05) of TRPV1 was recorded (4.95 ± 1.44). ANOVA followed by Dunnett's multiple comparison tests. Where, *NS* = p > 0.05; **p   <  0.01.

#### Molecular docking

**Figure 8:**
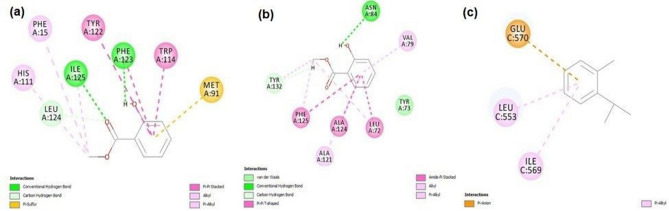
Interactions of betula oil (methyl salicylate), cinnamaldehyde and eugenol against. Odorant Binding Proteins (OBP) of *Aedes* (**a**), OBP of *Anopheles* (**b**), and TRPV1 proteins of rats (**c**). betula oil formed two conventional hydrogen bond interactions with Phe123 and Ile125; one carbon hydrogen bond interaction with Leu124; six hydrophobic interactions (P-Pi Stacked, Alkyl and Pi-Alkyl) with Phe15, His111, Trp114, Tyr122 and Ile125. O-Cymene formed two hydrophobic interactions with Leu553 and Ile569; and one Pi-Anion interaction with Glu570 of TRPV1 of rats. All these interacting amino acid residues are the key components of the reported active binding site of the selected targets.

From molecular docking studies, we found eight compounds, namely, betula oil, cinnamaldehyde, citronellal, citronellol, estragole, eugenol, methyl eugenol and o-cymene, that showed better tendency to bind with the active site of all the three selected target proteins (data available in Supplementary Table S3). But, in case of OBP of both mosquito species (*Aedes* and *Anopheles*), betula oil showed best results with—CDocker energy − 23.5487 kcal/mol and − 22.737 kcal/mol, respectively. Whereas, in case of TRPV1 of rats, o-cymene showed the best results with CDocker energy − 19.981 kcal/mol. Binding free energies of the best compounds against their respective targets are shown in Supplementary Table S4. The interactions of the three compounds against their respective targets were shown in Fig. 8, where we found that, betula oil (methyl salicylate) formed two conventional hydrogen bond interactions with Phe123 and Ile125; one carbon hydrogen bond interaction with Leu124; six hydrophobic interactions (P-Pi Stacked, Alkyl and Pi-Alkyl) with Phe15, His111, Trp114, Tyr122 and Ile125. Betula oil formed one conventional hydrogen bond interaction with Asn84; one carbon hydrogen bond interaction with Tyr132; seven hydrophobic interactions (Pi-Pi T-shaped), Amide-Pi stacked, Alkyl and Pi-Alkyl) with Leu72, Tyr73, Val79, Ala121, Ala124, Phe125 and Tyr132 with OBP of *Anopheles* species. O-Cymene formed two hydrophobic interactions with Leu553 and Ile569; and one Pi-Anion interaction with Glu570 of TRPV1 of rats. All these interacting amino acid residues are the key components of the reported active binding site of the selected targets. The interactions of the remaining compounds were given in supplementary materials (Supplementary Figs. S5–Figs. S7).

## Discussion

Synthetic repellents are always problematic and have negative public perceptions due to their side effects and toxicity to users as well as harmful effects to ecology and non-target organisms^38–40^. EOs are rich in terpenoids and effective against mosquitoes due to their insecticidal and repellent property. They are economic, safe, easily available, less toxic, and decrease the chances of resistance to insects due to their complex chemistry. In our previous research, we reported the attraction and repellency assay of fourteen EOs against *Musca domestica* using ‘Y’-tube olfactometer. The blend of clove, citronella and lemongrass oil exhibited maximum flight orientation response. In this study also, the blend of citronella, clove and lemon grass oil showed best results against mosquitoes under K&D study. The efficacy of EO depends on the different phytoconstituents present in each oil.

Under characterization, FT-IR study confirmed the EOs and other auxiliary formulation ingredients are compatible to each other and peaks of the physical mixture are intact and correlated to the individual chemicals. TGA thermogram showed the weight loss rates of EOs as a function of temperature elevation from 30 to 600 ℃. Onset weight loss of clove oil was reported as 80–90 ℃^41^ previously. In our study, EO-MRC showed first weight loss region from 50 to 260 °C, which might be due to the removal of loaded EO molecules with water contents from the repellent cream bases. The second weight loss region was evidenced from 270 to 600 °C, which might be due to the decomposition of excipients used to formulate EO-MRC. Similar study was carried out by Sattary et al.; prepared a lemongrass oil and clove oil encapsulated antifungal mesoporous silica nanoparticles against wheat’s take-all disease^42^. GC–MS was utilized for identification of the chemical compositions present in Eos and also to evaluate the assay of eugenol and citronellol present in EO-MRC. For identification and quantitation of volatile as well as semi-volatile compounds, GC–MS provided high throughput and high sensitivity of analytical results^43^.

Kaplan–Meier survival function was previously utilized by Gray et al.; to determine daily mortality of pyrethroid resistant *Ae. aegypti* for each day of post-insecticide exposure^44^. To measure the fraction of subjects living or survived or protected after treatment, Kaplan–Meier estimate is one of the best options to be utilized^45^. In this study, mosquito bioassay was carried out to determine CPT as per standard test guidelines, recommended by WHO^46^. Efficacy of EOs have been previously reported by Azeem et al.^47^. They revealed more than 45 min of protection time under arm in cage bioassay^47^. Our study results confirm that, after processing the best blend of EOs into formulations, the developed product gave complete protection by up to 228 min, respectively.

Lipophilic molecules present in EOs might have a direct entry into the lung cells of the user as there is only one cell membrane to pass through. For which, the effect of breathing EO fragrances from EO-MRC can be considerable. Chances of toxicity to the lungs might be there due to excessive use of EO, but the actual concentration has not been worked out and very few studies are available^48^. The Flavour and Extract Manufacturers Association (FEMA) granted generally recognized as safe (GRAS) status to most of the EOs and they are approved by the US Food and Drug Administration (US FDA) for utilization in food, cosmetics and pharmaceutical products. After doing evaluation by the expert panel of the FEMA, this was reviewed in 1996. Basically, most flavour ingredients at less than 100 ppm can be used for exposure, and predictions regarding their safety can be assessed^49^. Thus, from our study results on L-132, EO-MRC might be considered appropriate for effective mosquito repellent without producing any harmful effect to the lung cells.

Determination of acute dermal irritation study is useful where formulation to be exposed in skin. It provides information on health hazards likely to arise from a short-term exposure by the dermal route^50^. Acute dermal irritation study of EO-MRC on rabbit and repeated dose dermal toxicity study on Wistar rats confirms that EO-MRC is safe and do not produce any harmful effects to the skin. Under acute eye irritation study, EO-MRC proved its non-irritative nature to any ocular tissues. A test substance is categorized as United Nations Globally Harmonized System of Classification and Labelling of Chemicals (UN GHS) eye irritant category 2A when it shows a positive response of conjunctival redness ≥ 2; conjunctival edema ≥ 2 corneal opacity ≥ 1; iritis ≥ 1; estimated as the mean scores after grading in different time intervals as 24 h, 48 h and 72 h following instillation of the test substance, and which entirely reverses within an observation time of 21 days. If the above-mentioned effects are entirely reversible within an observation time of 7 days, than the test substance would be categorized under category 2B^51^.

Synthetic pyrethroids and organophosphates are becoming a threat to fish and other aquatic animals^52^. Hedayati et al., reported hematological and gill histopathology data on iridescent shark (*Pangasius hypophthalmus*) exposed to organophosphate and pyrethroid insecticides^53^. In our study, non-target toxicity on *D. rerio* confirms non-toxic nature of EO-MRC. Here also, DLM was used as negative control. The Fish gill histopathology of EO-MRC was found to be same tissue architecture as the un-treated (control) gills. Our finding on *D. rerio* gill has similar results as previously reported on fish gill histopathology.

A decreased AChE was determined in rats brain exposed to TNSF, which might be associated in increase in lipid peroxidation^54^, and possibility of disruption of metabolic and nervous activities^55^. However, EO-MRC exposed rats did not show any significant difference with the control rats. Increased activity of AChE causes a decreased amount of ACh, which causes a reduction in blood flow and vasodilatation^56^. In brain hypoperfusion by ligating common carotid arteries, reduction in ACh in the hippocampal area is responsible for memory and learning impairment^57^. In various research studies, the loss of perivascular cholinergic terminals has been reported in Alzheimer’s disease patients^58,59^. Moreover, AChE inhibitor medication is known to affect cholinergic function in subjects treated with hypobaric hypoxia (↓AChE activity, ↑ACh levels and upregulation of choline acetyltransferase, enzyme that has a role in ACh formation) and eventually memory function^60^.

Odorant binding proteins (OBPs) are the first relay in semi-chemical reception in insects as they are the liaison between the air medium that broadcasts chemical signals and odorant receptors which are located in olfactory structures (mainly the antenna and maxillary pulps) of insect’s peripheral sensory system^61^. No significant expression of OBP-2A in EO-MRC exposed rats head part have been seen, however, mosquitoes exposed to EO-MRV, containing the same EOs showed overexpression of OBP-2A. It might be due to the exposure pattern of EO to the target groups. It confirms that, EOs have the capability to overexpress OBP of mosquitoes. In case of synthetic TNSF exposed mosquito head part, OBP-2A was inhibited. In case of TRPV1, EO-MRV exposed mosquito head part showed overexpression of this protein, where EO-MRC exposed group did not show any changes and TNSF exposed group showed inhibition of TRPV1 respectively. This might be an indication of TRPV1 being expressed more in inflamed tissues, thereby associating its significance in the antennal inflammatory cascade of mosquitoes. Same results were observed in case of anti-OBP2A antibody also. This is the first report that demonstrates the expression of TRPV1 and anti-OBP2A antibody in mosquito head part. Insect use at least three families of olfactory receptors, viz., Odorant Receptors (ORs), Ionotropic Receptors (IRs) and Gustatory Receptors (GRs)^12,62^. DEET masks the olfactory receptor neurons (ORNs) to attractants and decreases the sensitivity to lactic acid, a human sweat component by decreasing the response of lactic acid excited neurons and increasing the inhibition of lactic acid inhibited neurons^12^.

EO-MRC showed very promising results against mosquitoes. WB analysis supported the significant expression of the target proteins when treated with developed formulation containing the EOs as active ingredients. Hence, in the in-silico study, we tried to find out the exact molecules present in the EOs responsible for the repellent activity. From the in silico docking study, eight compounds were identified which showed better affinity to bind with the target proteins of the mosquitoes and rat species to form stable protein–ligand complex. The in silico study revealed that the eight identified compounds of the EOs play significant role in the overall repellency property of the developed product.

## Materials and methods

### Chemicals and reagents

EOs of basil (*Ocimum basilicum* L.), bergamot (*Citrus bergamia* Risso & Poit), camphor [*Cinnamomum camphora* (L.) J. Presl.], cinnamon (*Cinnamomum zeylanicum* Blume), citronella [*Cymbopogon nardus* (L.) Rendle], clove (*Eugenia caryophyllus* Wight), eucalyptus (*Eucalyptus globulus* Labill.), jasmine (*Jasminum officinale* L.), lavender (*Lavandula angustifolia* Mill.), lemon grass [*Cymbopogan citratus* (DC.) Stapf], mentha (*Mentha piperita* L.), rosemary (*Rosmarinus officinalis* L.), patchouli (*Pogostemon patchouli* Benth), and wild turmeric (*Curcuma aromatica* Salisb.) were procured from Talent Technologies (Talent Technologies, Kanpur, India). Acetylcholinesterase (AChE) activity assay kit, Anti-OBP2A antibody, ELISA kits, 1,1-diphenyl-2-picrylhydrazyl (DPPH), radioimmunoprecipitation (RIPA) buffer and phosphate buffer saline (PBS) were purchased from Sigma Aldrich (Sigma Aldrich Chemical Co., St. Luis, USA). TRPV1 antibody was purchased from Santa Cruz (Santa Cruz, California, USA). 1-chloro-2,4-dinitrobenzene (CDNB) was purchased from Cayman (Cayman Chemical Company, Michigan, USA). Human normal lung cell line (L-132) was obtained from the National Centre for Cell Sciences (NCCS), Pune, India. High performance liquid chromatography (HPLC) grade acetone was purchased from Merck (Merck Pvt. Ltd., Mumbai, India). All other chemicals used were of the highest analytical grade available.

### Test insects

5–7 days old adult female *Ae. albopictus* mosquitoes were housed at the laboratory insectary, Division of Pharmaceutical Technology, Defence Research Laboratory, Tezpur, Assam, India. Mosquitoes were reared by maintaining temperature at 27 ± 2 °C, relative humidity: 75 ± 5% RH and 14L:10D h of light–dark alternative cycles in standard-sized wooden cages (75 cm × 60 cm × 60 cm) with a sleeve opening on one side as described previously^63^. 10% sucrose solution ad libitum were provided for nourishment*.* Before testing, the mosquitoes were starved for 24 h.

### Screening of EOs

Dose response study was performed to evaluate the best oils among the fourteen EOs. This study was approved (approval number: 032/2021TMCH, 28/08/2018) by the Institutional Human Ethical Committee (IHEC), of the Tezpur Medical College & Hospital (TMCH), Tezpur, Assam, India, and all experiments were performed in accordance with relevant guidelines and regulations. Five volunteers are chosen, not allergic to mosquito bite and all volunteers provided written informed consent. A volunteer’s thigh was marked according to the door opening hole of the K&D module as described by Klun and Debboun^64^. It is made of Plexiglas and the base of the rectangular cage (26 cm × 5 cm × 5 cm) has six holes, each with rectangular 3 × 4 cm holes that are opened and closed by a sliding door (Supplementary Fig. S8: Provide the photograph of K&D module). The flexor region of the forearms of a human volunteer was outlined with four rectangular (3 cm × 4 cm) test areas. A volume of 25 µL of each concentration of the EOs in soybean oil (40, 4 and 0.4 µg/cm^2^) and 25 µL of the soybean oil (diluent) as control was applied to the marked areas. After air drying for 5 min, a K&D module with matching cut outs in its floor was placed over the treated areas, containing five nulliparous 5–7 days old female mosquitoes in each hole. The doors of the cells were opened and the number of mosquitoes biting in each cell was recorded within a 2 min exposure, after which the doors were closed. After completion of each observation, mosquitoes were freed by opening cells of the K&D module in a sleeved screened cage. For each test, fresh sets of mosquitoes are used. Five replications for each test were carried out. The efficacy of EOs were determined by the percentage repellency against mosquitoes, using the formula or Eq. (2) described by WHO^46^.

2 $$\% \;{\text{repellency}} = \frac{C - T}{C} \times 100$$

where, C is the number of mosquitoes landing, or biting at the control area; T is the number of mosquitoes landing or biting at the treated area.

### Fourier transform-infra red spectroscopy (FT-IR)

Study of chemical compatibility for each formulation ingredients are necessary. All formulation ingredients possess specific value of vibrational frequency and have varied functional groups in their chemical structures. For compatibility study, each EOs, excipients to be used in cream formulation, and their physical mixture was placed one by one over the sample plate of the FT-IR instrument (Bruker, ALPHA, Billerica, MA, USA). The covering probe was placed over the sample and IR spectra was obtained over a wavelength of 2.5–25 μm at room temperature. Functional groups possessed by each individual ingredient should be identical in their physical mixture which confirms their compatibility^37^.

### Thermogravimetric analysis (TGA)

The thermal behaviour of citronella oil, clove oil, lemon grass oil, their mixture and EO-MRC were evaluated using a thermal analyser (TG 209 F1 Libra^®^, NETZSCH-Gerätebau GmbH, 95100 Selb, Germany). Approximately about 10 mg sample weight was placed in the crucible each time. Nitrogen was used as a shielding gas. Heating program was fixed as 30–600 °C at a rate of 10 °C/min.

### Formulation development and optimization

For optimization, a 17-run, 3-factor, 3-level Box-Behnken design (BBD) was utilized. A second order polynomial model was constructed by quadratic response surface methodology (RSM) using Design-Expert software (Version 6.0.8, Stat-Ease Inc., USA). Total seventeen formulations were obtained using EO concentrations as dependent variables against complete protection time (CPT) as independent variable or response variable. Analysis of variance (ANOVA) was performed using the same software to obtain the most effective formulation.

### Preparation of cream

Phase inversion temperature method was applied for the preparation of EO-based mosquito repellent cream (EO-MRC). About 50 g cream sample was prepared in order to get enough for performing the various qualitative and quantitative assay. The oil phase (phase B) was prepared by dissolving the oil soluble excipients, except phase A (mosquito repellent active ingredients) under mild heating at 200 rpm in a hot magnetic plate stirrer (Magnetic Stirrer IKA RCT basic) and heated to 65 °C. The aqueous phase was prepared by mixing various aqueous soluble ingredients (phase C) under gentle heating and stirring. Temperature of the aqueous phase was raised to 65 °C. Phase A was gently added to the oil phase at a stirring speed of 200 rpm and 55 ± 2 °C. The mixture was then emulsified by adding phase C slowly and kept for 1 h at a stirring rate of 800 rpm and 60 ± 2 °C. The formulated EO-MRC was then kept for natural cooling.

### Efficacy assessment

CPT of the developed cream (EO-MRC) formulation was carried out by arm in cage bioassay. 1 mL EO-MRC was applied to ≈ 600 cm^2^ area of the forearm skin between the wrist and elbow and 1 mL of the 12% N, N-di ethyl benzamide (DEBA) based marketed cream (DBMC) was compared on the other arm. Two mosquito cages (size: 40 × 40 × 40 cm) each containing 200–250 non-blood-fed female *Ae. Albopictus* were used. One cage is designated for testing the EO-MRC and the other for the positive control (DBMC). During testing, hands were protected by surgical gloves for which the mosquitoes cannot bite while the volunteer avoids movement of the arm. EO-MRC and DBMC treated arms were exposed for 3 min at 30 min intervals to determine landing and/or probing activity. A single landing or probing of mosquito within a 3 min test interval concludes the test. CPT was calculated as the time (min) required for the first mosquito landing or probing after repellent application to the treated area. The median CPT and confidence intervals were estimated from the Kaplan–Meier Survival Function^46^.

Efficacy was correlated with DEBA based marketed cream (DBMC). The inclusion of the specific commercial product DBMC is for comparison and does not constitute any recommendations.

### Characterization

#### Gas chromatography-mass spectroscopy (GC–MS)

#### Qualitative study

Different chemical components in fourteen EOs and the selected blend were identified by a GC–MS system of Agilent Technologies (5301 Stevens Creek Blvd. Santa Clara, CA 95051, United States). Test sample concentration of 500 μg/mL was prepared in GC grade acetone. A sample volume of 1 μL was introduced into the injector held at 250 °C. Oven temperature of 40–300 °C was programmed at 20 °C/min. Helium was used as carrier gas at flow rate 1 mL/min. The injector and detector temperature were set at 250 °C and 230 °C (quad) and 150 °C (core) respectively^37^. Standard C7–C30 saturated alkanes were purchased from Sigma Aldrich Chemicals Co., St. Louis, USA. Retention indices (RI) of the identified components were determined for identification of the detected components.

#### % Assay by GC–MS study

Calibration samples of eugenol and citronellol were prepared by dissolving an appropriate amount in GC grade acetone to get concentrations of 62.5 μg/mL, 125 μg/mL, 250 μg/mL and 500 μg/mL. Test samples of EO-MRC, clove oil and citronella oil were prepared by dissolving a required amount in acetone to quantify the EO components in the final formulation. A sample volume of 1 μL was introduced into the injector as described in ‘Qualitative study’ section.

#### Physicochemical parameters

Physical parameters of the EO-MRC and placebo formulations were determined in order to establish aesthetic compliance and consumer acceptability. To determine the viscosity, a programmable viscometer was used (Model: DV2T, Ametek Brookfield, Middleboro, MA, USA); combined with software Rheo3000, version 1.2.2019.1 [R]. Sample volume was fixed at 30 g and viscosities were determined at 10 rpm for 40 s at room temperature using a T-Bar spindle (B-92) (Helipath spindle set, Brookfield Engineering Labs. Inc). Density was determined by using a pycnometer. pH of EO-MRC was checked by using digital pH meter (Labman Scientific instruments, Tamil Nadu, India).

Spread ability of EO-MRC was determined as per the method reported earlier by Sabale^65^. In brief, 1 g of EO-MRC was placed on 1 cm^2^ pre-marked circular area on the glass slide (7.5 cm × 2.5 cm). EO-MRC was compressed using another glass slide placed from edge to centre of primary slide. 200 g of commercial weight was placed on the set up and allowed the gel to spread for the period of 1 min. The spread diameter was calculated with the aid of graph paper and spread ability was evaluated using formula expressed as Eq. (3):

3 $$\mathrm{Spread\, ability}=\mathrm{m}\times \frac{\mathrm{l}}{\mathrm{t}}$$

where, m is the commercial weight placed on the setup; l is the length of cream spread; and t is the time.

### Safety assessment

#### Cytotoxicity by MTT assay

The reduction of tetrazolium salts is now widely accepted as a reliable way to examine cell proliferation. The yellow tetrazolium MTT (3-(4,5-dimethylthiazolyl-2)-2,5-diphenyltetrazolium bromide) is reduced by metabolically active cells, in part by the action of dehydrogenase enzymes, to generate reducing equivalents such as NADH and NADPH. With the help of spectrophotometric means, the resulting intracellular purple formazan can be quantified. The assay measures the cell proliferation rate and conversely, when metabolic events cause apoptosis or necrosis, the reduction in cell viability^66^.

Cells cultured in T-25 flasks were trypsinized and aspirated into a 5 mL centrifuge tube. Cell pellet was obtained by centrifugation at 3000 rpm. The cell count was adjusted, using DMEM HG medium, such that 200 μL of suspension contained approximately 10,000 cells. To each well of the 96 well microtiter plate, 200 μL of the cell suspension was added and the plate was incubated at 37 ℃ and 5% CO_2_ atmosphere for 24 h. After 24 h, the spent medium was aspirated. 200 μL of different test concentrations viz. 62 µg/mL, 125 µg/mL, 250 µg/mL, 500 µg/mL, and 1000 µg/mL, of EO-MRC were added to the respective wells. The plate was then incubated at 37 °C and 5% CO_2_ atmosphere for 24 h. The plate was removed from the incubator and the drug containing media was aspirated. 200 μL of medium containing10% MTT reagent was then added to each well to get a final concentration of 0.5 mg/mL and the plate was incubated at 37 ℃ and 5% CO_2_ atmosphere for 3 h. Without disturbing the crystals formed in the wells, culture medium was completely removed. 100 μL of solubilisation solution (DMSO) was added to each well and the plate was then gently shake in a rocking shaker (ROCKYMAX™, Tarsons, Kolkata, India) to solubilize the formed formazan. The absorbance was measured at a wavelength of 570 nm and also at 630 nm using a microplate reader. The percentage growth inhibition was calculated and concentration of EO-MRC needed to inhibit cell growth by 50% (IC_50_) was generated from the dose–response curve for the cell line.

#### Animals and ethics statement

All experimenting protocols using animal were performed according to the “Principles of Laboratory Animal care” (NIH publication 85–23, revised 1985) and approved by the Institutional Animal Ethical Committee (IAEC) of Defence Research Laboratory (DRL), Tezpur, Assam, India (approval no. CPCSEA/DRL/Protocol no. 3, 20/06/2018). All studies involving animals are reported in accordance with the ARRIVE guidelines for reporting experiments involving animals^67^. All efforts were made during the study period to minimize the suffering of animals and to reduce the number of animals used.

5–8 weeks old, about 210–250 g of male healthy adult Wistar rats (*Rattus norvegicus*) and young and healthy New Zealand albino rabbits (*Oryctolagus cuniculus*) were obtained from the institutional animal housing facility and allowed to acclimatize for 7 days prior to the study. Standard food and purified water ad libitum were provided in clean and hygienic condition at 22–25 ℃, 40–70% RH with 12 h light–dark cycles.

#### Acute dermal irritation study

Acute dermal irritation study was conducted on healthy New Zealand albino rabbits following the OECD test guidelines 404^68^. Approximately 24 h before the test, fur was removed from the dorsal area of the trunk. 0.5 g EO-MRC, was directly applied to the skin and after 4 h exposure period, residual EO-MRC was removed by using water without disturbing the integrity of the epidermis and examined for signs of erythema and oedema, at 60 min, and then at 24 h, 48 h and 72 h after EO-MRC removal. Dermal reactions are graded and recorded according to the grades in the Table 8. As per the method described by Banerjee et al.^69^; primary irritation index (PII) was calculated. Further, we have followed the Draize method of classification for PII scoring as non-irritant (if PII < 0.5), slightly irritant (if PII < 2), moderately irritant (if PII ≤ 2–5), and severely irritant (if PII > 5)^70^ and then mean irritation score per time point was calculated. The mean scores at day 1, day 2 and day 3 were then summed up and followed the equation to obtain the PII.

$${\text{PII}} = {{\left( {\sum \left( {Xa + Xb} \right)t\_1 + \sum \left( {Xa + Xb} \right)t\_2 + \sum \left( {Xa + Xb} \right)t\_3} \right)} \mathord{\left/ {\vphantom {{\left( {\sum \left( {Xa + Xb} \right)t\_1 + \sum \left( {Xa + Xb} \right)t\_2 + \sum \left( {Xa + Xb} \right)t\_3} \right)} 3}} \right. \kern-\nulldelimiterspace} 3}$$

where, Xa is the mean score of erythema formation; Xb is the mean score of edema formation; $t\_1$ is the day 1; $t\_2$ is the day 2; $t\_3$ is the day 3.

**Table 8:** Grading of skin reactions.

Dermal reactions		Grading
Erythema and eschar formation	Oedema formation	
No erythema	No oedema	0
Very slight erythema (barely perceptible)	Very slight oedema (barely perceptible)	1
Well defined erythema	Slight oedema (edges of area well defined by definite raising)	2
Moderate to severe erythema	Moderate oedema (raised approximately 1 mm)	3
Severe erythema (beef redness) to eschar formation preventing grading of erythema	Severe oedema (raised more than 1 mm and extending beyond area of exposure)	4

#### Repeated dose dermal toxicity study

As per the OECD guideline 410^71^, the repeated dose dermal toxicity of the EO-MRC was carried out for a period of 21 days. Healthy Wistar rats were housed in 2 different groups (control, and EO-MRC treated) and each group contained 06 animals after initial acclimatization for at least 5 days prior to the study. Briefly, dorsal fur was removed using sterile surgeon hair removal blade (49–20 mm) and 4.0 × 4.0 dorsal area was treated with placebo and test substances for at least a 6 h/day on a 5 day/week basis, for 21 days. An observation with respect to body weight and feed consumption was monitored on a daily basis^72^.

#### Acute eye irritation

For this experiment, OECD TG 405, acute eye irritation testing procedure was carried out on young and healthy rabbits^50^. 50 mg EO-MRC was placed to the conjunctival sac for 5 s by gently pulling the lower lid of the right eyeball; where, left eye was served as control. Capsaicin, an ocular irritant was used as negative control. The eye of the animal was not washed for the next 24 h following installation of capsaicin. Observations for any ocular lesions at specific intervals of 1 h, 24 h, and 48 h were evaluated using slit lamp microscope (Haagstreit type AIA-11, Appaswamy)^73^.

### Non-target toxicity testing

#### Acute toxicity test on zebrafish (*Danio rerio*)

The acute toxicity test on *Danio rerio* (*D. rerio*) was carried out in accordance with the guidelines of the Organization for Economic Cooperation and Development (Test no. 203, Acute Immobilization Test)^74^. Seven neonate *D. rerio* were exposed in each test vessel and three replicates were tested, for a total of 21 *D. rerio* per treatment group. *D. rerio* individuals were placed in containers with 250 mL of pure water, and EO-MRC was diluted with acetone and mixed into the water in dosages corresponding to the concentrations 200, 100 and 50 mg/L. The experiment was divided into three groups. The first two groups were formed by *D. rerio* individuals, exposed to the action of EO-MRC and negative control deltamethrin (DLM) for a period of 24 h^75^. *D. rerio* individuals in the third group was served as control.

The physical and chemical conditions of the acute toxicity tests were as follows: pH ranging from 7.2 to 7.6; constant temperature of 25 ± 1 °C; electrical conductivity around 160 μS/cm; dissolved oxygen above 3 mg/L. The number of dead *D. rerio* in the three replicates was counted and used to determine the LC_50_ for 24 h of exposure.

#### Acetylcholinesterase (AChE) activity assay

In this study, transfluthrin-1.6% (TNSF) and EO-MRC were exposed to different rat groups (n = 6) for 21 days. Control animals received no exposure. After completion of 24 h from the last exposure, all animals were humanely sacrificed by cervical dislocation. Brain tissue samples were collected and homogenized using 0.1 M phosphate buffer, pH 7.5, followed by centrifugation at 14,000 rpm for 5 min. Cleared supernatants were used for assay, as per the procedure described in the technical bulletin of Acetylcholinesterase activity assay kit (Sigma-Aldrich, St. Louis, MO 63103 USA; Catalog Number: MAK119).

#### Protein estimation in mosquito head part

Head part of female *Ae. albopictus* (n = 100 in each group) previously exposed to EO-MRV (essential oil-based mosquito repellent vaporizer), EO-MRC, TNSF and control (un-treated) were isolated and freshly homogenized in RIPA buffer and centrifuged at 12,000 rpm for 15 min. Collected supernatants were kept at − 80 °C for further utilization. As per the instructions mentioned in the Biorad DC Protein assay protocol (Bio-Rad, Hercules, CA), protein concentrations in the tissue samples were determined. Working reagents and protein standard dilutions were prepared to construct the protein standard curve. 5 μL of standards and tissue samples were placed in appropriate wells, followed by 25 μL and 200 μL of working reagents A and B respectively. The absorbance was read at 750 nm after 15 min using a microplate reader (Spinco Biotech Pvt. Ltd., India). From the results of this assay, the amount of protein in each test sample equivalent to 50 μg standard protein was calculated for further loading in the blotting process.

#### Western blotting

Required amount of test samples was mixed with an equal amount of the Laemmli Buffer and used for blotting. Sodium dodecyl sulphate polyacrylamide gel electrophoresis (SDS-PAGE) gels were prepared from the 10% acrylamide resolving gel (TGX Stain-FreeTM FastCastTM Acrylamide Kit, Bio-Rad, CA, US) as per manufacturer’s instructions. Standard protein markers (Precision Plus, Kaleidoscope, Biorad) and previously prepared tissue samples were introduced into different wells of the casted SDS-PAGE gels. The electrophoresis was carried out in a Mini-PROTEAN® Tetra System and PowerPacTM HC electrophoresis power supply system (Bio-Rad, CA, US). After electrophoresis, the protein embedded gel was transferred at 15 mV (15 min) to a nitrocellulose membrane in the Trans Blot Turbo machine. The protein bands embedded membrane was then transferred to a fresh petri plate and incubated on a rocking shaker (Rockymax, Tarsons, Kolkata, India) for 1 h in a blocking solution consisting of Tris buffer saline (TBS) with 0.1% Tween 20 and 5% bovine serum albumin (BSA). After 1 h, the blocking solution was discarded and the membrane washed three times (3 min/wash) with TBST solution. The TBST solution was completely pipetted out and without drying the membranes, specifically diluted primary antibodies were added to the membranes. The membranes were probed separately with anti-OBP2A and TRPV1 and β-actin primary antibodies (diluted in 5% BSA in Tris-buffered saline, pH 7.4) and incubated overnight at 4 °C. The next day, the membrane was washed in TBST 3 times (5 min/wash), followed by 1 h incubation with horseradish peroxidase (HRP) linked secondary antibodies (diluted in 5% BSA in Tris-buffered saline, pH 7.4, (details mentioned in Supplementary Table S5) on rocking shaker at 4 °C. After incubation, freshly prepared ECL substrate was added to the membranes and immediately visualized and interpreted in G: Box Chemi-XRQ gel doc system (Syngene, United Kingdom). The intensity of bands was calculated with the SynGene GeneTools (SynGene Laboratories, Cambridge, United Kingdom). Results are representative of three independent experiments. All WB band quantification steps were performed using custom ImageJ (National Institutes of Health; http://rsb.info.nih.gov/ij/) script.

### In silico screening of the EO components

#### Molecular docking and MM-PBSA (Molecular Mechanics Poisson-Boltzmann Surface Area) based binding free energy calculation

Molecular docking study was carried out to find the component(s) of the EOs which have a tendency to bind with the target(s) associated with repellent activity. The components of EOs used in the formulation were identified from the GC–MS analysis. Then the SMILE id of the compounds was retrieved from PubChem database (https://pubchem.ncbi.nlm.nih.gov/) and loaded to the Discovery Studio 2020 molecular modelling software (DS 2020) (Dassault BIOVIA, San Diego, USA)^76^. The 3D structures of the compounds were generated and energy minimization of the compounds was performed according to standard protocol of DS 2020 using smart minimizer method^77^. The target proteins odorant binding protein (OBP) for *Aedes* species (PDB: 3K1E) and for Anopheles species (PDB: 3QME), and TRPV1 for rat (PDB: 5IS0) were downloaded from protein data bank (www.rcsb.org)^78^. The target proteins were cleaned, prepared and then energy minimized using the ‘smart minimizer’ method for 2000 steps with energy RMSD gradient 0.01 kcal/mol^77^. After that the binding sites for docking study were selected using the information given in Protein Data Bank for active sites of the selected targets. The active sites for OBP of *Aedes* species was X: 13.63, Y: 40.63, Z: 24.92 and radius 9.8 Å; for OBP of Anopheles species was X: 20.17, Y: 31.50, Z: 32.09 and radius 7.7 Å; for TRPV1 of rats was X: 107.77, Y: 92.80, Z: 103.16 and radius 9.3 Å (Supplementary Fig. S4). Then docking study was carried out using simulation-based docking protocol CDocker of DS 2020 which provides comparatively more accurate information regarding the binding of a compound in the active site^79^. The best docking poses generated were also analysed to observe the different non-bonding interactions took place between the target proteins and compounds.

The MM-PBSA based binding free energy provides accurate information regarding the thermodynamic stability of protein–ligand complex in real physiological conditions^80,81^. Hence the best poses of the compounds selected from docking were further analysed to calculate the binding free energies (ΔG) using MM-PBSA method.

## Conclusion

The present study investigates the repellent activity of a non-toxic EO-MRC formulation against mosquitoes, as per the international standards. EO-MRC tested herein, has successfully passed the efficacy study against *Ae. albopictus* and preclinical toxicity on different laboratory animals as per OECD test guidelines. The developed product provides effective protection up to 228 min, against *Ae. albopictus* without producing any health hazards in preclinical settings. Thus, the overall study substantiates the development of EO based, longer lasting, non-toxic topical cream formulation to strategically minimize mosquito bites and to decrease the mosquito-borne disease incidents. However, this study is not conclusive, further study on repellency testing using different strains of mosquito, with different bioassay procedures like tunnel bioassay, y-tube olfactometer etc., should be carried out. Non-target toxicity testing, stability testing or self-life assessment of the EO-MRC is essential for more conclusive results. In future, multiple emulsion and/or liposomal cream formulation could be designed for controlled release of anti-mosquito formulations in exposed skin to attain maximum protection time. This would reduce the dosing frequency in outdoor conditions. Study on expression of more odour responsive protein available in the mosquito head part might contribute towards the scientific community in understanding more detailed mechanism of repellent chemical. The present report will benefit research students, formulation scientists and pharmaceutical companies to develop effective, safe and longer lasting herbal mosquito repellents that could be applicable to travellers, the military in jungle operations or the general public in mosquito endemic areas to get protection from mosquitoes.

## Supplementary Information

Supplementary Information.
