# Supplementary material for: The fabrication and assessment of mosquito repellent cream for outdoor protection
Source: Sci Rep. 2022 Feb 9;12:2180. doi: 10.1038/s41598-022-06185-9 (PMC8828860; doi:10.1038/s41598-022-06185-9)
Supplement: Supplementary file 1 — Supplementary Information. [file 41598_2022_6185_MOESM1_ESM.docx]

**Supplementary File**

Development of mosquito repellent cream for outdoor protection: fabrication, bio-efficacy, safety and in silico screening

## Hemanga Hazarika^1,2,5*^, Harshita Krishnatreyya^1,2^, Varun Tyagi^3^, Johirul Islam^4^, Neelutpal Gogoi^5^, Danswrang Goyary^1^, Pronobesh Chattopadhyay^1*^, Kamaruz Zaman^5^

*^1^Division of Pharmaceutical Technology, Defence Research Laboratory, Tezpur-784001, Assam, India*

*^2^Girijananda Chowdhury Institute of Pharmaceutical Science, Dekargaon, Tezpur-784001, Assam, India*

*^3^Eurofins Agroscience Services Pvt. Ltd., Tirupur-641603, Tamil Nadu, India*

*^4^Coromandel Int. Ltd., Shameerpet-500101, Telangana, India*

*^5^Department of Pharmaceutical Sciences, Dibrugarh University, Dibrugarh-786004, Assam, India*

### ***Corresponding authors**

Dr. Pronobesh Chattopadhyay

Division of Pharmaceutical Technology,

Defence Research Laboratory, Tezpur-784001, Assam, India

Tel: +91-3712258498, E-mail: chattopadhyay.drl@gmail.com

Hemanga Hazarika

Division of Pharmaceutical Technology,

Defence Research Laboratory, Tezpur-784001, Assam, India

Tel: +91-8638096366, E-mail: hemanga14@gmail.com

**Supplementary Tables**

**Supplementary Table S1:** Physical parameters of EO-MRC and placebo formulation

| Parameters | EO-MRC | Placebo formulation |
| --- | --- | --- |
| Color | White | White |
| Odor | Aromatic and pleasant* | Characteristic** |
| Spradability | Excellent and uniform | Uniform |
| Density (g/mL) | 1.02±0.01 | 1.04±0.01 |
| Viscosity(centipoise) | 28878.33±594.99 | 30775±770.55 |
| Torque (%) | 62.6±3.48 | 76.8±4.14 |
| pH | 7.3±0.08 | 6.93±0.13 |
| Spreadability | 24.3±0.63 | 21.66±0.83 |

*Aromatic and pleasant odor refers to the fragrance of essential oils which gives freshness and soothes the human olfactory senses

**Characteristic odor refers the familiar odor of excipients, and that which does not smell of rancidity.

**Supplementary Table S2:** Acute dermal irritation study of EO-MRC and its placebo in rabbits

| Positive control (0.8%w/v aqueous solution of formaldehyde) (n=6) | | | | | | |
| --- | --- | --- | --- | --- | --- | --- |
| Dermal reactions | Erythema | | | Oedema | | |
| Observation time (days) | 1 | 2 | 3 | 1 | 2 | 3 |
| Total score | 21 | 18 | 17 | 13 | 13 | 11 |
| Mean score | 3.5 | 3 | 2.83 | 2.16 | 2.16 | 1.83 |
| Total mean score | 15.48 | | | | | |
| PII | 5.16 | | | | | |
| Remarks | Severely irritating | | | | | |
| Negative control (placebo EO-MRC formulation) (n=6) | | | | | | |
| Dermal reactions | Erythema | | | Oedema | | |
| Observation time (days) | 1 | 2 | 3 | 1 | 2 | 3 |
| Total score | 0 | 0 | 0 | 0 | 0 | 0 |
| Mean score | 0 | 0 | 0 | 0 | 0 | 0 |
| Total mean score | 0 | | | | | |
| PII | 0 | | | | | |
| Remarks | Non irritating | | | | | |
| Treated group (EO-MRC formulation) (n=6) | | | | | | |
| Dermal reactions | Erythema | | | Oedema | | |
| Observation time (days) | 1 | 2 | 3 | 1 | 2 | 3 |
| Total score | 0 | 0 | 0 | 0 | 0 | 0 |
| Mean score | 0 | 0 | 0 | 0 | 0 | 0 |
| Total mean score | 0 | | | | | |
| PII | 0 | | | | | |
| Remarks | Non irritating | | | | | |

**Supplementary Table S3:** Docking results of the best eight compounds.

| Name | CDocker Energy (kcal/mol) | | |
| --- | --- | --- | --- |
|  | Anti-OBP2A of *Aedes* species | Anti-OBP2A of *Anopheles* species | TRPV1 of rats |
| Betula oil | -23.5487 | -22.737 | -18.056 |
| Cinnamaldehyde | -15.0927 | -18.0465 | -16.3491 |
| Citronellal | -7.52582 | -9.87592 | -2.58623 |
| Citronellol | -5.59067 | -5.62655 | -0.927337 |
| Estragole | -15.4979 | -9.50012 | -7.51936 |
| Eugenol | -18.5536 | -11.1556 | -15.635 |
| Methyleugenol | -13.6415 | -4.40126 | -9.66778 |
| o-Cymene | -21.253 | -11.312 | -19.9812 |

**Supplementary Table S4:** Binding free energies of the best compounds against their respective targets.

| Target | Compound | Binding free energy (kcal/mol) |
| --- | --- | --- |
| OBP of *Aedes* | Betula oil | -15.7321 |
| OBP of *Anopheles* | Betula oil | -6.7499 |
| TRV1 of rats | o-Cymene | -3.0587 |

**Supplementary Table S5:** Details of the primary and secondary antibodies used in WB

| Primary antibody | Secondary antibody |
| --- | --- |
| TRPV1(mouse monoclonal IgG; 1:500) | Goat anti-mouse IgG-HRP (1:5000) |
| Anti-OBP2A (rabbit monoclonal IgG; 1:100) | Anti-rabbit IgG-Peroxidase (1:10000) |

**Supplementary Figures**

**
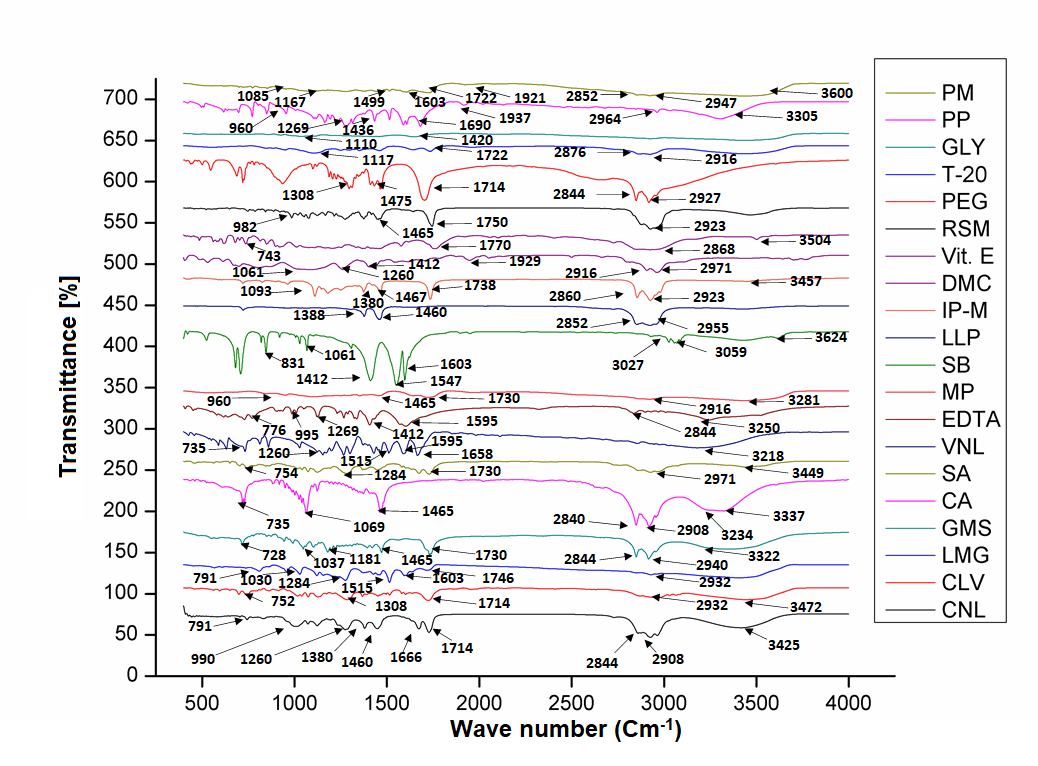
**

**Supplementary Figure S1:** FT-IR spectroscopical analysis of different formulation ingredients along with their physical mixture. Where, CNL: citronella oil; CLV: clove oil; LMG: lemon grass oil; GMS: glycerol myristate; CA: cetyl alcohol; SA: stearic acid; VNL: vanillin; EDTA: Ethylenediaminetetraacetic acid; MP: methyl parabean; SB: sodium benzoate; LLP: light liquid paraffin; IP-M: isopropyl myristate; DMC: dimethicone; Vit. E: vitamin E; RSM: rosemary oil; PEG: polyethylene glycol; T-20: tween 20; GLY: glycerine; PP: propyl paraben; PM: physical mixture


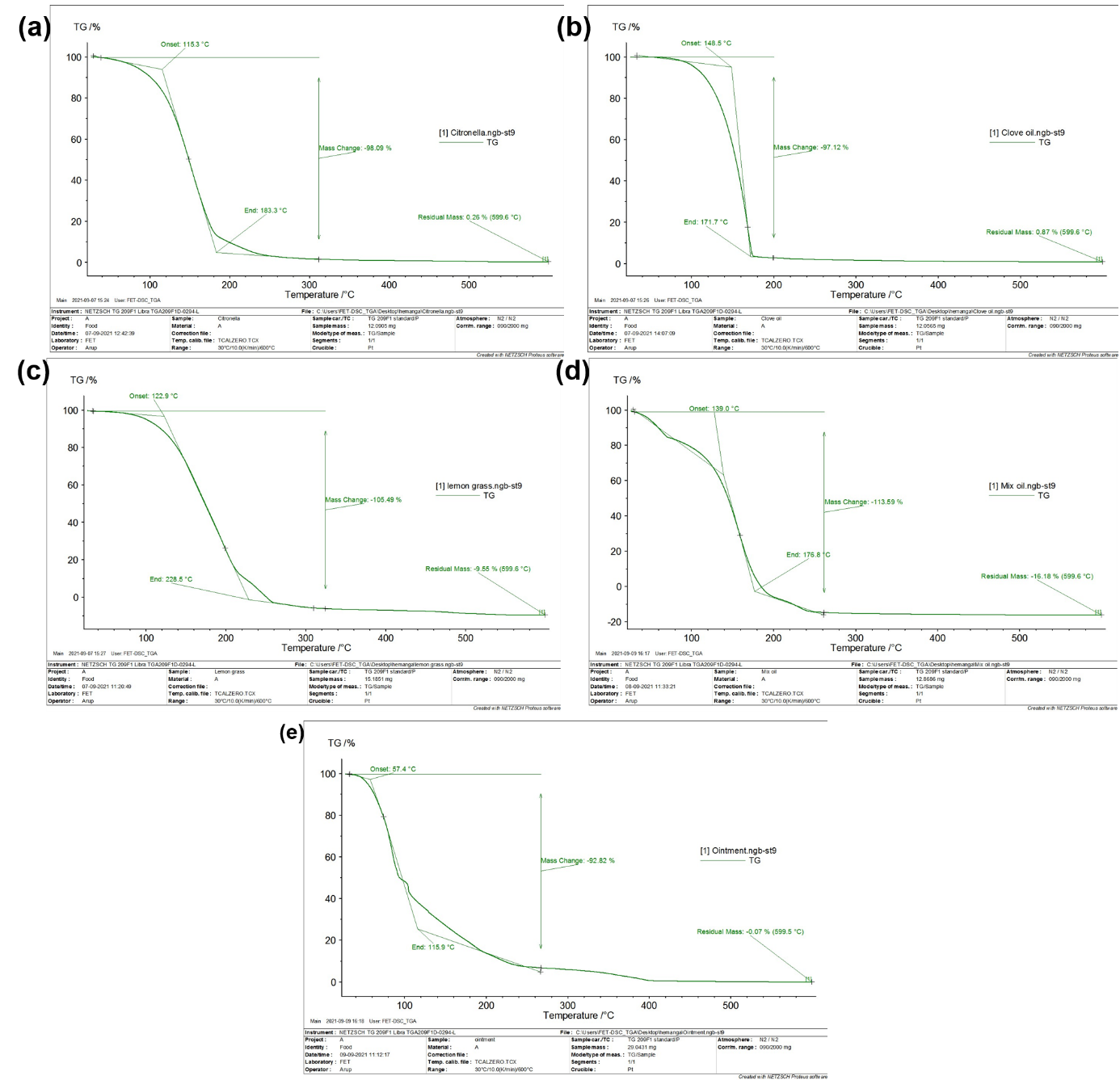


**Supplementary Figure S2:** Thermogravimetric analysis of (a) citronella oil; (b) clove oil; (c) lemon grass oil; (d) physical mixture of citronella oil, clove oil and lemon grass oil; and (e) EO-MRC


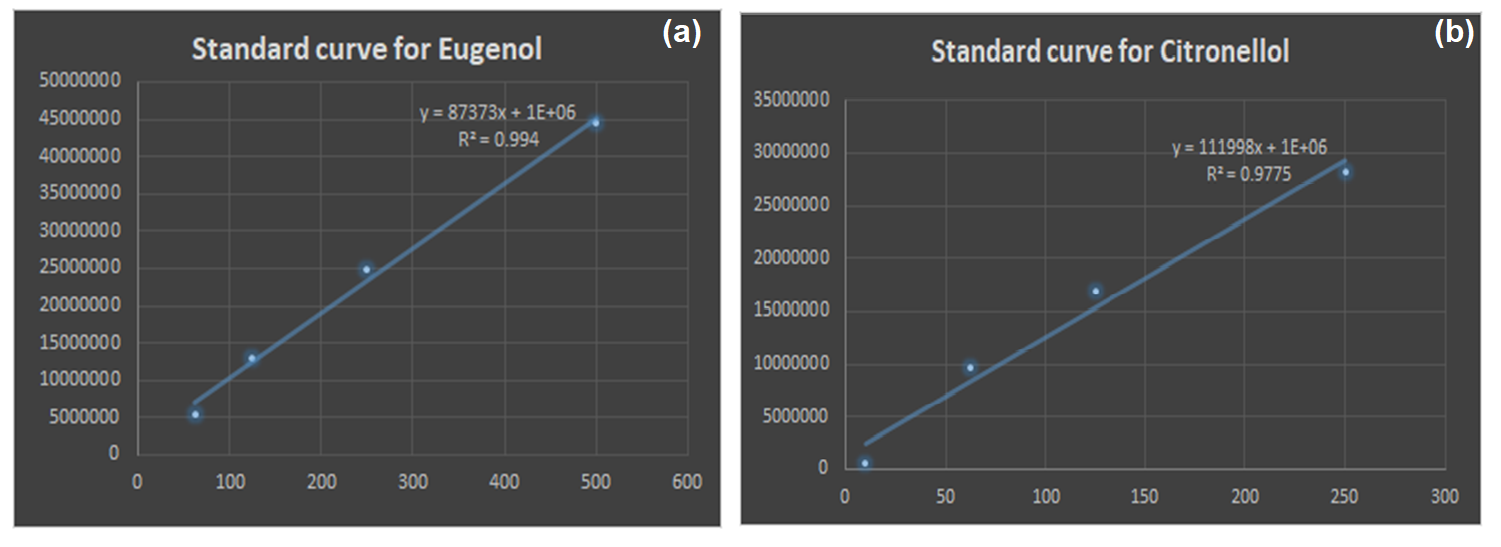


**Supplementary Figure S3:** Standard calibration curve for(a) eugenol and (b) citronellol


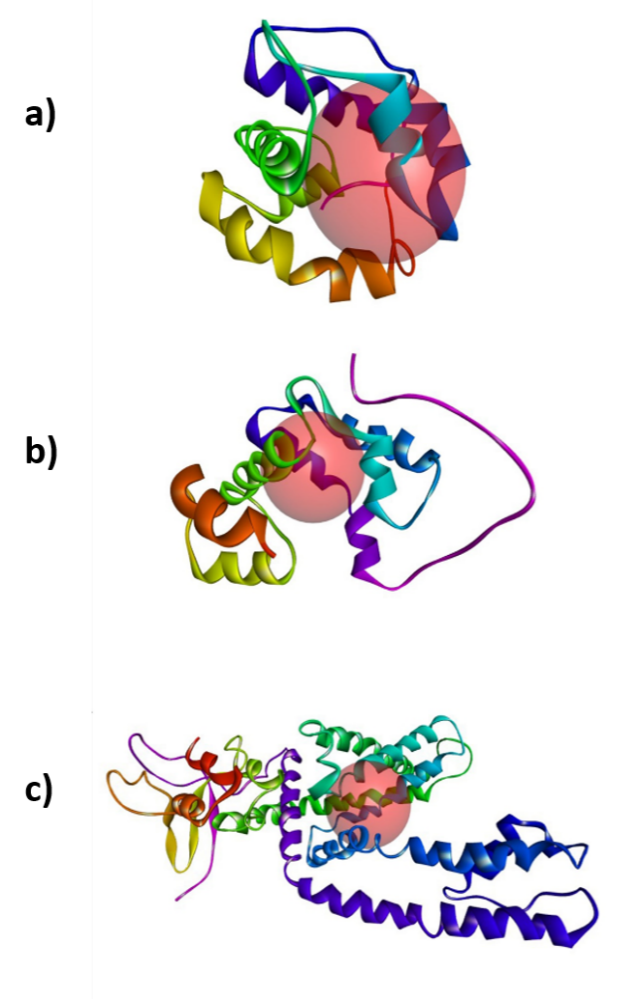


**Supplementary Figure S4:** Active binding sites of the target proteins; a) OBP of Aedes species, b) OBP of Anopheles species and c) TRPV1 of rat


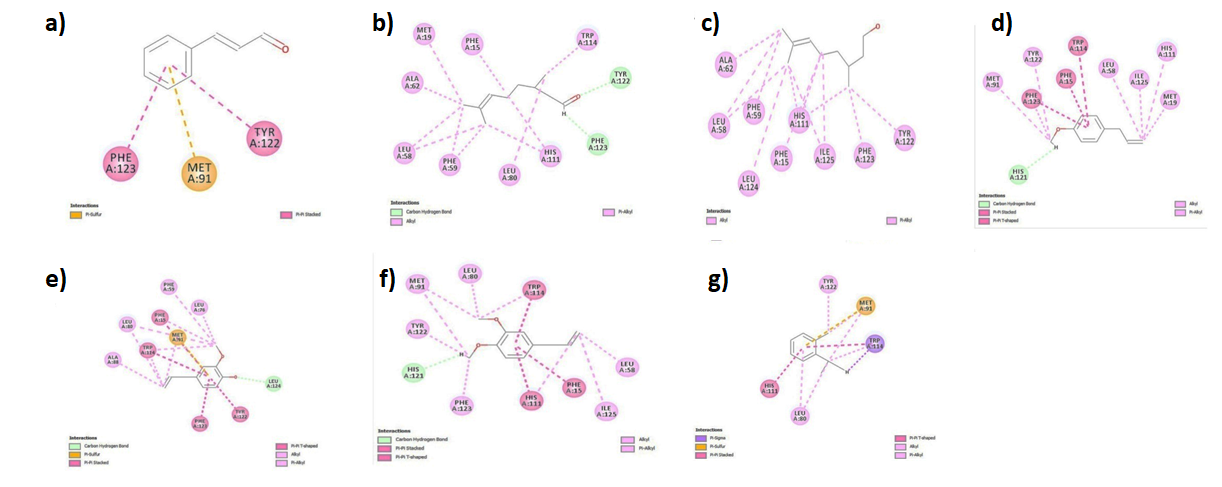


**Supplementary Figure S5:** Compounds showing good binding affinity with OBP of *Aedes* species a) Cinnamaldehyde, b) Citronellal, c) Citronellol, d) Estragole, e) Eugenol, f) Methyleugenol and g) o-Cymene


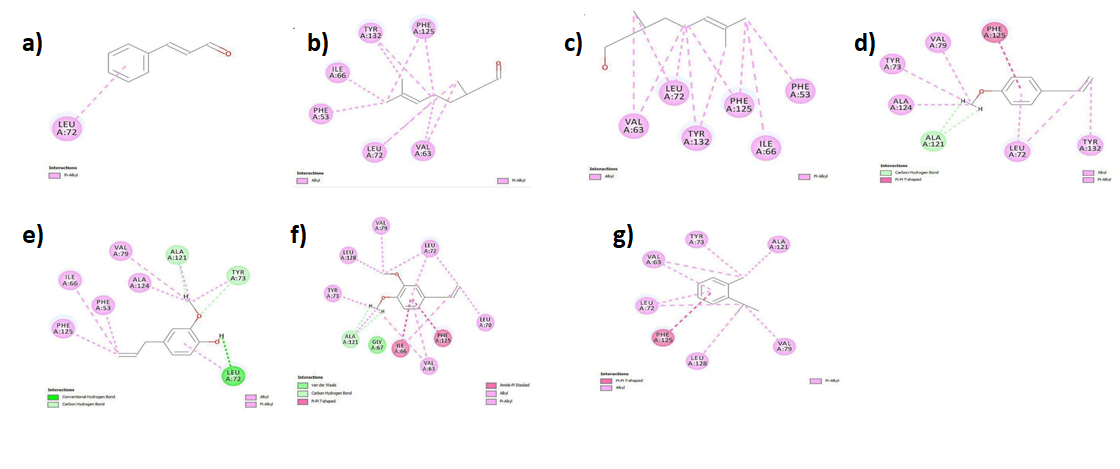


**Supplementary Figure S6:** Compounds showing good binding affinity with OBP of *Anopheles* species a) Cinnamaldehyde, b) Citronellal, c) Citronellol, d) Estragole, e) Eugenol, f) Methyleugenol and g) o-Cymene


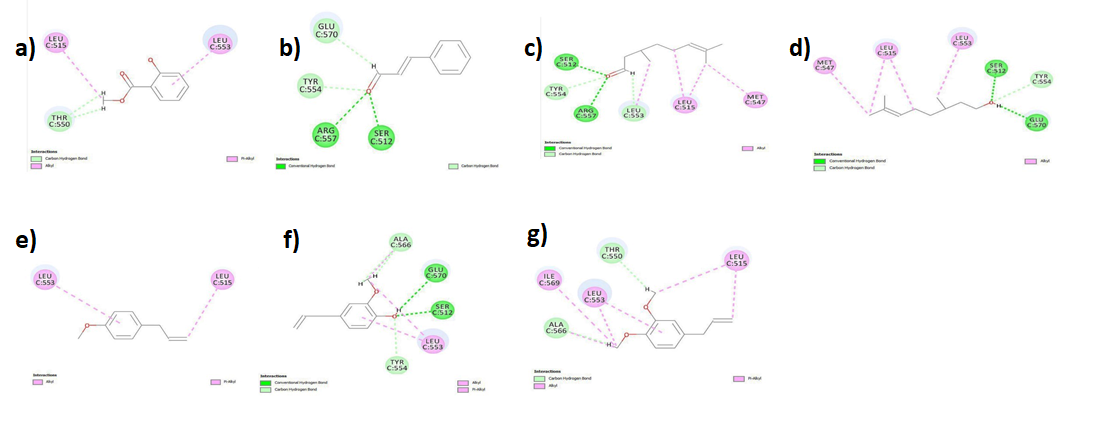


**Supplementary Figure S7:** Compounds showing good binding affinity with TRPV1 of rats. a) Betula oil, b) Cinnamaldehyde, c) Citronellal, d) Citronellol, e) Estragole, f) Eugenol and g) Methyleugenol


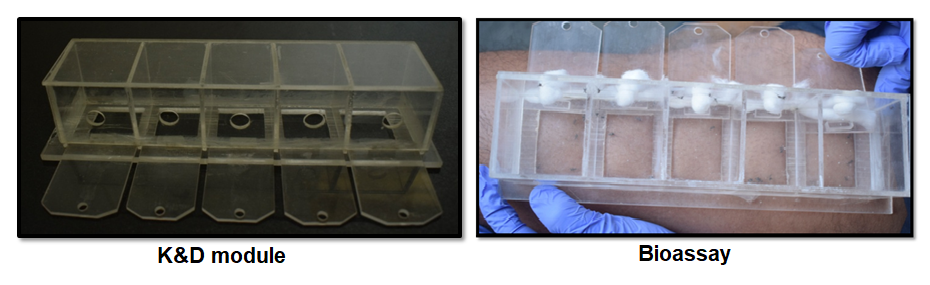


**Supplementary Figure S8:** K&D module for mosquito bioassay


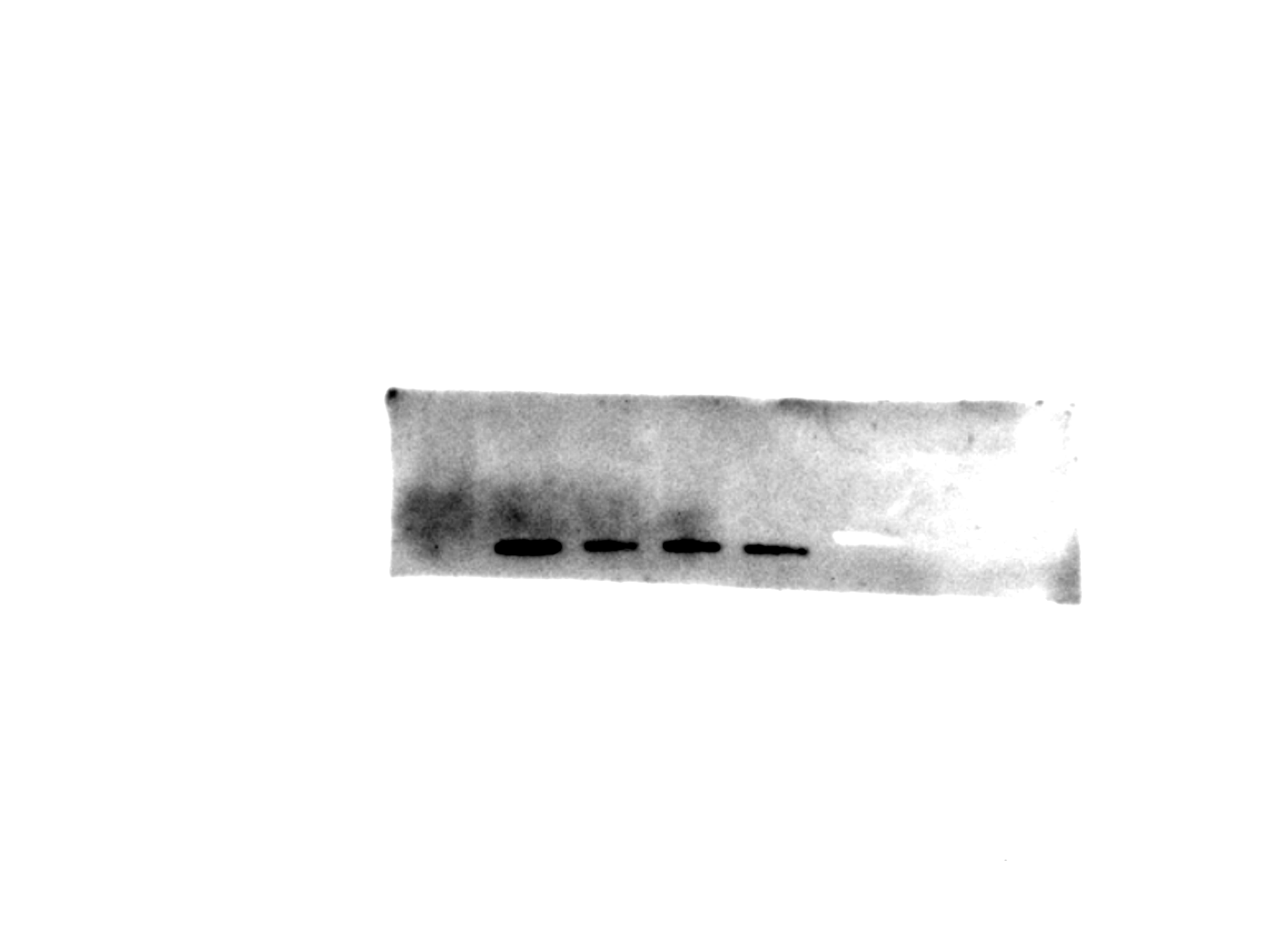


42kDa

**Supplementary Figure S9:** Full-length blots/gels of β actin


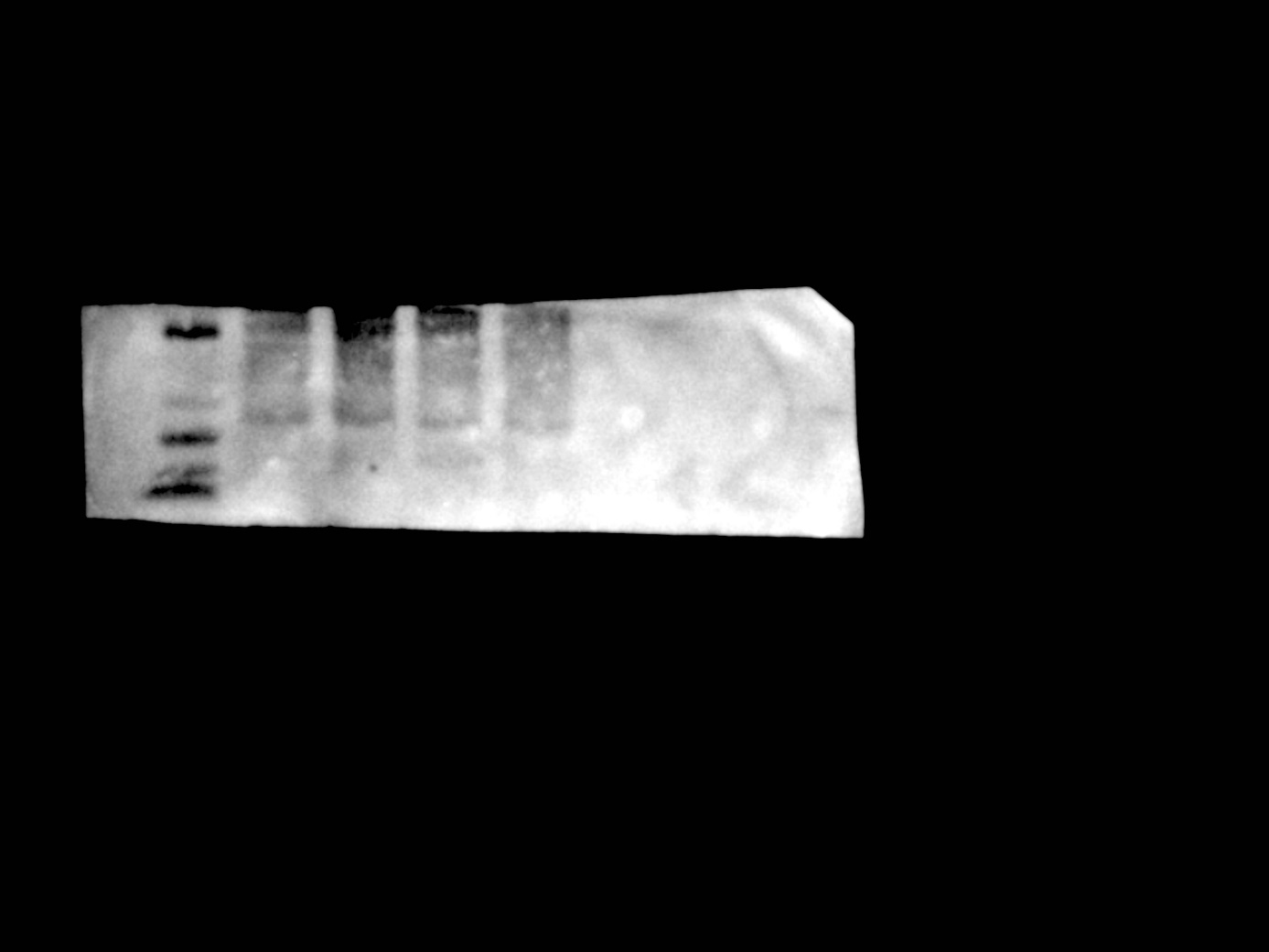


22kDa

**Supplementary Figure S10:** Full-length blots/gels of Anti-OBP2A


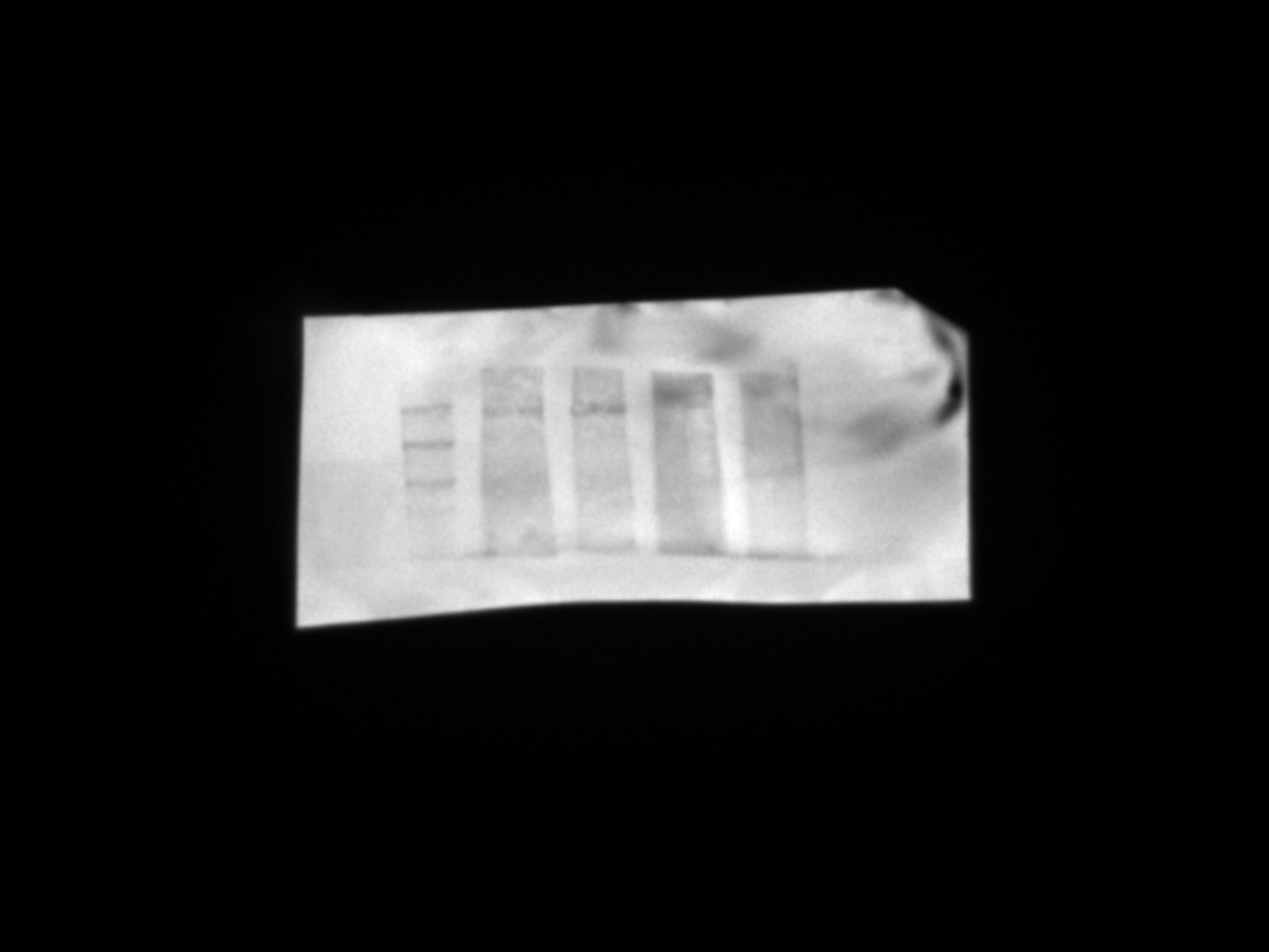


100kDa

**Supplementary Figure S11:** Full-length blots/gels of TRPV1
